# Chd4 and ThPOK cooperate to preserve structural and electrophysiological integrity of the adult heart through Sprr1a repression

**DOI:** 10.1111/febs.70583

**Published:** 2026-05-15

**Authors:** Fadoua El Abdellaoui‐Soussi, Apolonia Novillo, Rocío Brea, Marta Ysbert, Lorena Cussó, Gonzalo Pizarro‐Sánchez, Manuel Desco, Iván Hernández, Ángel Luis Armesilla, Ignacio Pérez de Castro, Alicia R. Folgueras, Juan Miguel Redondo, Pablo Gómez‐del Arco

**Affiliations:** ^1^ Institute for Rare Diseases Research, Instituto de Salud Carlos III (ISCIII) Madrid Spain; ^2^ Gene Regulation in Cardiovascular Remodelling and Inflammation Laboratory Centro Nacional de Investigaciones Cardiovasculares (CNIC) Madrid Spain; ^3^ Departamento de Biología Celular e Histología, Facultad de Medicina Universidad Complutense de Madrid (UCM) Spain; ^4^ Unidad de Imagen Avanzada Centro Nacional de Investigaciones Cardiovasculares (CNIC) Madrid Spain; ^5^ Unidad de Medicina y Cirugía Experimental Hospital General Universitario Gregorio Marañón, Instituto de Investigación Sanitaria Gregorio Marañón (IiSGM) Madrid Spain; ^6^ CIBER de Salud Mental Instituto de Salud Carlos III Madrid Spain; ^7^ Cardiology Department, Ruber Juan Bravo Hospital and Faculty of Medicine, Health, and Sports Universidad Europea de Madrid Spain; ^8^ Translational Laboratory for Cardiovascular Imaging and Therapy Centro Nacional de Investigaciones Cardiovasculares Carlos III (CNIC) Madrid Spain; ^9^ Centro de Investigación Biomédica en Red de Enfermedades Cardiovasculares (CIBERCV) Madrid Spain; ^10^ Departamento de Bioingeniería Universidad Carlos III de Madrid Spain; ^11^ Facultad HM de Ciencias de la Salud Universidad Camilo José Cela Madrid Spain; ^12^ Instituto de Investigación Sanitaria HM Hospitales Madrid Spain; ^13^ Departamento de Bioquímica y Biología Molecular, Facultad de Medicina Instituto Universitario de Oncología del Principado de Asturias (IUOPA), Universidad de Oviedo Spain; ^14^ Instituto de Investigación Sanitaria del Principado de Asturias (ISPA) Oviedo Spain; ^15^ Cell‐Cell Communication & Inflammation Unit, Centro de Biología Molecular Severo Ochoa (CBMSO) Consejo Superior de Investigaciones Científicas‐Universidad Autónoma de Madrid Spain; ^16^ Present address: Center for Stem Cells and Organoid Medicine (CuSTOM) Cincinnati Children's Hospital Medical Center Cincinnati OH USA

**Keywords:** arrhythmia, cardiac conduction system, cardiomyocyte differentiation, Chd4, heart, laminopathy, nuclear lamina, Sprr1a, ThPOK

## Abstract

The structural and electrophysiological stability of the adult heart depends on coordinated transcriptional repression governed by chromatin remodeling and nuclear architecture. The Chd4/NuRD complex is essential to maintain cardiomyocyte identity, and its deletion leads to arrhythmias, aberrant expression of skeletal muscle sarcomeric genes, and dysregulation of key components of the cardiac conduction system, including *Hcn4*, *Cntn2*, and *Cx40*. However, while the mechanisms by which Chd4 cooperates with additional transcriptional regulators to preserve adult cardiac homeostasis remain poorly understood, previous studies have identified ThPOK, a lamina‐associated transcription factor, as a candidate Chd4 partner in cardiomyocytes. Using cardiomyocyte‐specific conditional mouse models, we show that combined loss of *Chd4* and *ThPOK* markedly exacerbates conduction defects, leading to more severe arrhythmias and accelerated sudden cardiac death compared with *Chd4* loss alone. Transcriptomic profiling revealed a synergistic upregulation of Sprr1a, a protein normally expressed in the epidermis where it contributes to keratinocyte differentiation and cornification. *Sprr1a* induction correlated with downregulation of the cardioprotective *microRNA miR‐150‐5p*, a change driven by *Chd4* deletion; however, *miR‐150‐5p* levels were not further reduced in double‐knockout hearts, indicating that ThPOK contributes to *Sprr1a* de‐repression through a distinct mechanism. Notably, *Sprr1a* upregulation was also observed in *Lmna* mutant hearts, suggesting a link between its de‐repression and cardiac‐specific nuclear lamina dysfunction. Together, these findings uncover a cooperative Chd4;ThPOK regulatory axis that preserves transcriptional integrity and safeguards structural and electrical homeostasis in the adult murine heart.

## Graphical abstract

Chd4/NuRD and ThPOK cooperate to maintain transcriptional repression and nuclear organization in adult cardiomyocytes. *Chd4* loss reduces *miR‐150‐5p*, relieving repression of *Sprr1a*, while ThPOK loss further enhances Sprr1a activation, possibly through altered chromatin–lamina interactions. Therefore, combined *Chd4* and *ThPOK* deficiency leads to strong *Sprr1a* upregulation and severe arrhythmias. Similarly, disruption of nuclear lamina integrity in *Lmna* ^*R249W*^ mutant hearts is also associated with increased *Sprr1a* expression.

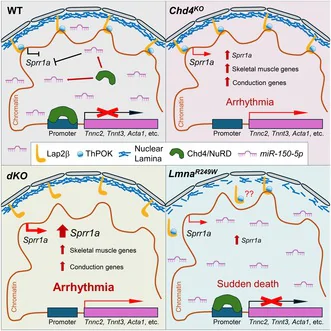

**Abbreviations**
Acta1: Actin, Alpha 1, Skeletal Muscle
AF: Atrial Fibrillation
CCS: Cardiac Conduction System
Chd4: Chromodomain‐Helicase‐DNA‐binding protein 4
Cntn2: Contactin 2
*dKO*
: *Chd4;ThPOK* double knockout
ECG: Electrocardiogram
EF: LV Ejection Fraction
FS: LV fractional shortening
GATA4: GATA‐binding protein 4
Hcn4: hyperpolarization‐activated cyclic nucleotide‐gated potassium channel 4
HF: Heart Failure
IHC: Immunohistochemistry
IVRT: Isovolumetric Relaxation Time
LAD: Lamina‐Associated Domain
Lmna: Lamin A
LV Vol;d: Diastolic Left Ventricular Volume
LV Vol;s: Systolic Left Ventricular Volume
LVID;d: left ventricular LV internal diameter in diastole
LVID;s: left ventricular LV internal diameter in systole
MCK: Muscle Creatine Kinase
Myh11: Myosin Heavy Chain 11
Nkx2‐5: NK2 Homeobox 5
NuRD: Nucleosome Remodeling and Deacetylase
Smyd1: SET and MYND domain‐containing 1
Sprr1a: Small Proline‐Rich Protein 1A
Tbx5: T‐box transcription factor 5
TF: Transcription Factor
ThPOK: T‐helper‐inducing POZ/Krüppel‐like factor
VCS: Ventricular Conduction System
VT: Ventricular Tachycardia
WGA: Wheat Germ Agglutinin
ZMPSTE24: zinc metallopeptidase STE24
Znf219: Zinc Finger Protein 219

## Introduction

The precise regulation of gene expression in adult cardiomyocytes is essential for maintaining cardiac physiological function, including coordinated contraction, relaxation, and effective blood circulation throughout the organism. While the genetic architecture underlying cardiac function has been extensively characterized, the epigenetic mechanisms that orchestrate transcriptional control in cardiomyocytes remain comparatively underexplored, despite their potential impact on disease susceptibility and progression [1]. This gap in knowledge is particularly relevant in the context of cardiac arrhythmias, a heterogeneous group of disorders that affect millions of individuals worldwide and represent a major cause of heart failure, stroke, and sudden cardiac death. Beyond alterations in ion channels or structural proteins, growing evidence indicates that transcriptional dysregulation and loss of cardiomyocyte identity can profoundly impact electrical stability and myocardial function. Understanding how epigenetic regulators preserve appropriate gene expression programs in the adult heart is therefore critical for elucidating the molecular basis of arrhythmogenesis and for identifying new regulatory pathways that safeguard cardiac homeostasis [2].

Among the epigenetic regulators, chromatin remodelers play a pivotal role in modulating gene expression by altering chromatin structure and accessibility, thereby influencing cellular identity and function. The Chd4/NuRD chromatin remodeling complex is essential for maintaining cardiac muscle identity by repressing the expression of skeletal muscle sarcomeric genes in both developing and terminally differentiated cardiomyocytes [3, 4]. The loss of the helicase Chd4 releases this repression, leading to the formation of ‘hybrid myocytes’ that aberrantly express both cardiac and skeletal muscle genes, resulting in impaired cardiac function, arrhythmias, and ventricular dysfunction [3].

During embryonic heart development, Chd4 is recruited by transcription factors such as Gata4, Nkx2‐5, and Tbx5 to repress noncardiac gene programs in cardiomyocytes, including genes such as *Acta1* and *Myh11*, which encode skeletal and smooth muscle contractile proteins, respectively. In addition, Chd4 cooperates with the methyltransferase Smyd1 to repress metabolic and developmental pathways related to glycolysis, hypoxia response, and angiogenesis during cardiac maturation [5, 6]. In the adult heart, Chd4 interacts with the TF Znf219 to repress the skeletal muscle sarcomeric program [7]. These genes are normally expressed in skeletal muscle but actively silenced in cardiomyocytes to preserve cardiac‐specific contractile identity. Therefore, disruption of these pathways may result in the aberrant expression of skeletal muscle sarcomeric proteins in the heart, leading to arrhythmias and compromised myocardial contraction.

ThPOK (also known as cKrox), a transcription factor encoded by the *Zbtb7b* gene, physically interacts with the NuRD complex in lymphocytes to transcriptionally regulate CD4^+^ T‐cell differentiation [8]. In line with this, our *in silico* analysis also revealed a potential functional interaction between ThPOK and NuRD components in cardiomyocytes [7]. While ThPOK has been extensively studied in the immune system for its role in regulating T‐cell differentiation [9, 10, 11], it also regulates collagen expression in human fibroblasts [12] and lipid metabolism in mammary glands during late pregnancy and lactation [13]. Although its role in the adult heart has not been explored, ThPOK has recently been shown to contribute to the maintenance of heterochromatin organization during cardiomyocyte differentiation by tethering specific genomic loci to the nuclear lamina, thereby preserving transcriptional repression [14, 15]. This process could be important for the differentiation of cardiac progenitor cells and the transition of embryonic stem cells into cardiomyocytes, dependent on BMP4 signaling [16]. Given that lamina‐associated gene silencing is critical for stabilizing cell identity and that Chd4/NuRD plays a central role in repressing noncardiac gene programs in cardiomyocytes, these observations raise the possibility that ThPOK cooperates with Chd4/NuRD to maintain transcriptional homeostasis in the adult myocardium. However, whether such cooperation occurs in differentiated cardiomyocytes and whether it contributes to the preservation of cardiac electrical and structural integrity remains unknown.

In this study, we investigated the functional interplay between Chd4 and ThPOK in maintaining cardiac homeostasis in differentiated cardiomyocytes. Using cardiomyocyte‐specific single and double conditional knockout mouse models, we show that loss of *ThPOK* alone has minimal impact on cardiac structure and function, whereas its combined deletion with Chd4 markedly aggravates the arrhythmic phenotype and accelerates sudden cardiac death. Through integrative transcriptomic and phenotypic analyses, we identify dysregulation of genes involved in the cardiac conduction system and uncover a synergistic derepression of *Sprr1a*, a gene normally expressed in the epidermis but ectopically activated in mutant cardiomyocytes. We further show that *Chd4* loss leads to downregulation of the cardioprotective microRNA *miR‐150‐5p* [17, 18], while the additional contribution of ThPOK to *Sprr1a* derepression is independent of *miR‐150‐5p* and may correlate with alterations in nuclear lamina‐associated gene regulation. Together, our findings define a cooperative Chd4/ThPOK regulatory axis that integrates chromatin remodeling and nuclear architecture to preserve transcriptional integrity and electrical stability in the adult heart.

## Results

### Phenotypic characterization of *Chd4*, *ThPOK* and *Chd4;ThPOK* conditional KOs

*In silico* experiments and previous *in vivo* reports have suggested that TFs, such as Znf219 and ThPOK, interact with Chd4 in cardiomyocytes and T cells to regulate transcriptional programs, particularly through chromatin remodeling mechanisms [7, 8]. Given the limited information on ThPOK expression in the mammalian heart, we first assessed *ThPOK* mRNA expression during developmental stages and after birth (Fig. 1A) and across adult mouse tissues (Fig. 1B). During development, *ThPOK* transcript levels remained relatively stable between embryonic day (E) 10.5 and E18.5, with a modest reduction between E13.5 and E14.5, continuing into postnatal life (Fig. 1A). In 8‐week‐old mice, *ThPOK* expression was highest in the liver and testis, followed by skeletal muscle, thymus, spleen, and heart (Fig. 1B).

**Fig. 1:**
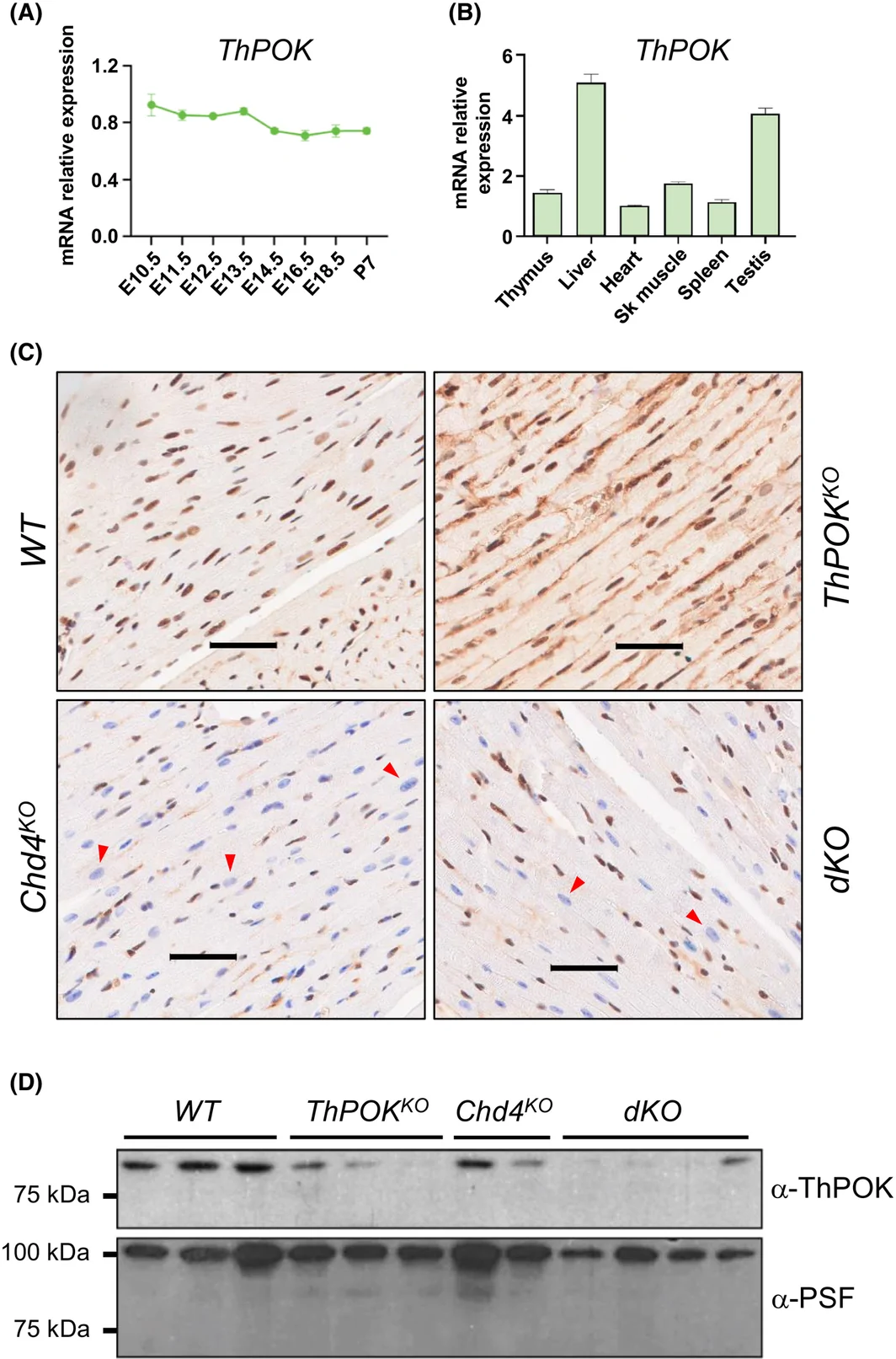
Chd4 and ThPOK expression in the hearts of WT and conditional mutant mice. (A) Relative *ThPOK* mRNA expression during mouse cardiac development (*n* = 3; mean ± SEM). (B) *ThPOK* mRNA levels in different adult mouse tissues (*n* = 4 mean ± SEM). Sk muscle: skeletal muscle (tibialis anterior). (C) Immunohistochemical (IHC) staining of Chd4 expression in representative transverse heart sections from *WT*, *ThPOK* ^*KO*^, *Chd4* ^*KO*^, and *dKO* hearts. All images were captured at 20 × magnification (scale bar = 100 μm). Red arrowheads show cardiomyocyte nuclei free of Chd4 protein. (D) Western blot analysis of whole‐heart protein extracts showing ThPOK (α‐ThPOK) expression in the same experimental groups as in (C). PSF (α‐PSF) was used as a loading control. All mice used in these experiments were male.

To examine the role of ThPOK in cardiac function, we generated cardiomyocyte‐specific *ThPOK* conditional KO (*ThPOK* ^*KO*^) mice. These mice, in which exons 2 and 3 of *Zbtb7b* were flanked by loxP sites [19] were crossed with transgenic mice expressing Cre recombinase under the muscle creatine kinase (*MCK*) promoter [20], leading to the deletion of *ThPOK* in postnatal terminally differentiated cardiomyocytes. Additionally, we generated *Chd4* conditional KOs [21] (*Chd4* ^*KO*^) and *Chd4;ThPOK* double mutants (*dKO*) to investigate the potential cooperative functions of these proteins.

To confirm efficient ablation of Chd4 and ThPOK, we performed immunohistochemistry (IHC) on cardiac cross sections to assess Chd4 expression, and Western blotting on whole‐heart extracts to evaluate ThPOK expression. Chd4 protein was localized to cardiomyocyte nuclei in wild‐type (WT) and *ThPOK* ^*KO*^ mice but undetectable in *Chd4* ^*KO*^ or *dKO* cardiomyocytes, while remaining detectable in noncardiomyocyte populations (Fig. 1C). Additionally, Western blotting confirmed a marked reduction of ThPOK protein in *ThPOK* ^*KO*^ and *dKO* hearts as compared to WT (Fig. 1D).

To evaluate the phenotypic consequences of these genetic deletions, we monitored survival rates among all genotypes. WT and *ThPOK* ^*KO*^ mice showed normal lifespans with no premature mortality. In contrast, *Chd4* ^*KO*^ and *dKO* animals exhibited increased mortality shortly after birth (Fig. 2A). Mortality in *Chd4* ^*KO*^ mice began around postnatal day 20, with 50% surviving until approximately Day 70. Strikingly, *dKO* mice exhibited more pronounced early mortality, with only 50% surviving until Day 50. Notably, while 20% of *Chd4* ^*KO*^ mice survived beyond 120 days, no *dKO* mice reached this age. These differences in mortality rate were statistically significant, indicating that ThPOK cooperates with Chd4/NuRD in mechanisms essential for cardiac homeostasis, as revealed by the markedly more severe phenotype in the double deletion.

**Fig. 2:**
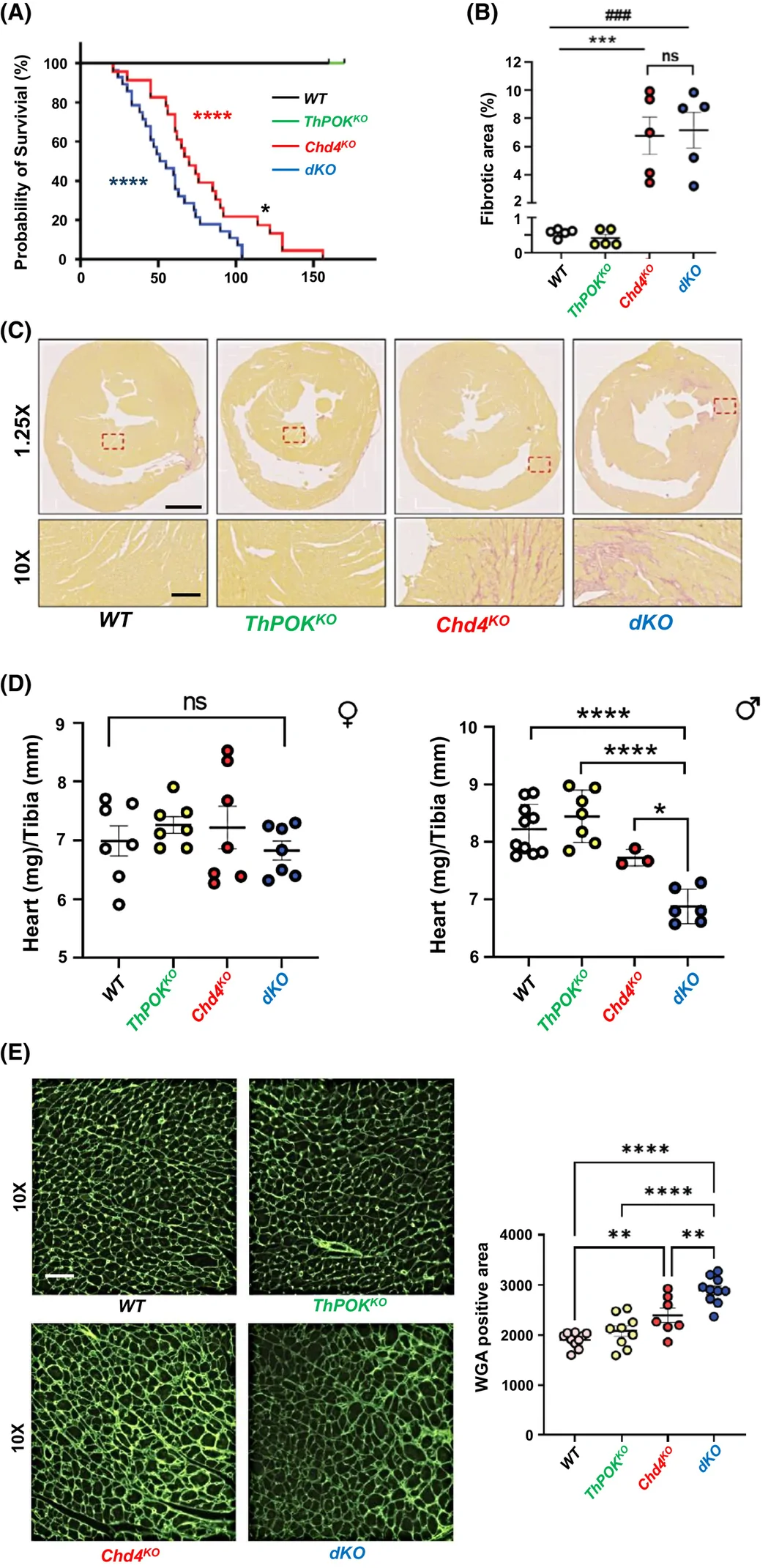
Phenotypic characterization of *Chd4* and *ThPOK* mutant mice. (A) Kaplan–Meier survival curves comparing *WT* (black line), *ThPOK* ^*KO*^ (green), *Chd4* ^*KO*^ (red) and *dKO* (blue) mice. Statistically significant differences were observed between *WT* and *Chd4* ^*KO*^ (red asterisks), *WT* and *dKO* (blue asterisks) and among *Chd4* ^*KO*^ *and dKO* mice groups (black asterisk). These statistical differences were assessed using the Log‐rank (Mantel‐Cox) test. *P‐value < 0.05 (0.021); ****P‐value < 0.0001. *WT* (*n* = 30), *ThPOK* ^*KO*^ (*n* = 30), *Chd4* ^*KO*^ (*n* = 23) and *dKO* (n = 27). In each experimental group, approximately 50% of the mice were male and 50% were female. (B) quantification of interstitial fibrosis in *WT* and mutant mice. *Chd4* ^*KO*^ and *dKO* hearts show a significant increase in interstitial collagen deposition. (C) representative images of Picrosirius Red staining in male heart sections from *WT*, *ThPOK* ^*KO*^, *Chd4* ^*KO*^, and *dKO* groups. Scale bars represent 1 mm (upper whole heart images) and 100 μm (lower 10 × magnifications). (D) Heart weight‐to‐tibia length ratios for females (left) and males (right), showing only *dKO* males a significantly reduced heart mass. (E) Left: WGA staining of transverse sections from male hearts (scale bar = 100 μm); the scale bar depicted in the WT image are also valid for the corresponding mutant pictures. Right: Quantification of cardiomyocyte cross‐sectional area. Significance for B, D, and E was determined using unpaired two‐sided t‐test (mean ± SEM). **P‐value <0.01; ****P‐value < 0.0001.

Given the exacerbated mortality in *dKO* animals, we further investigated potential underlying causes. Previous work has shown that Chd4 deficiency leads to cardiomyopathy, fibrosis, arrhythmia, and compromised cardiac function, ultimately resulting in sudden death [3]. Since fibrosis is a common feature in heart failure and hypertrophic cardiomyopathy, we assessed extracellular matrix deposition in young mutant hearts (Figs. 2B,C). As expected, *Chd4* ^*KO*^ hearts exhibited increased collagen deposition as compared to WT [3]. However, quantification revealed no additional fibrosis in *dKO* hearts as compared to *Chd4* ^*KO*^ hearts (Fig. 2B).

Cardiomyopathies often involve hypertrophic remodeling, which can be assessed by comparing heart weight to body size. We therefore analyzed heart weight normalized to tibia length, stratified by sex. No hypertrophy was observed in any group. However, male *dKO* mice showed significant heart hypotrophy (Fig. 2D). Because this reduction in heart size was only detected in males, we next focused our assessment of potential compensatory hypertrophy on male cardiomyocytes, evaluating their cross‐sectional area using wheat germ agglutinin (WGA) staining. Cardiomyocytes from male *Chd4* ^*KO*^ and *dKO* mice were indeed significantly enlarged as compared to WT and *ThPOK* ^*KO*^ littermates. Notably, cardiomyocyte hypertrophy was more pronounced in *dKO* males, consistent with a compensatory response to increased cardiomyocyte loss (Fig. 2E).

### *Chd4;ThPOK* double KO male mice exhibit impaired left ventricular systolic function, diastolic dysfunction and severe arrhythmias

To further elucidate the physiological consequences of the simultaneous deletion of *Chd4* and *ThPOK* in the heart, we conducted a longitudinal echocardiographic analysis comparing *Chd4* ^*KO*^, *ThPOK* ^*KO*^, and *dKO* mice with WT counterparts. To assess the progression of cardiac dysfunction, echocardiographic measurements were obtained at 5 and 10 weeks of age; these time points were selected because most mutants survive up to 5 weeks, allowing the detection of early cardiac abnormalities, whereas approximately 50% survive up to 10 weeks, enabling the identification of more pronounced defects at later stages. Given the sex‐related differences observed in the hypertrophic analyses (Fig. 2), mice were also analyzed separately by sex to evaluate potential sex‐dependent cardiac phenotypes.

We evaluated systolic functions by measuring the left ventricular LV internal diameter in diastole (LVID;d) and in systole (LVID;s), LV ejection fraction (EF), and fractional shortening (FS). We also measured diastolic and systolic LV volumes (LV Vol;d and LV Vol;s) (Fig. 3A). Additional structural, diastolic, and right ventricular parameters were measured and reported in Table S1.

**Fig. 3:**
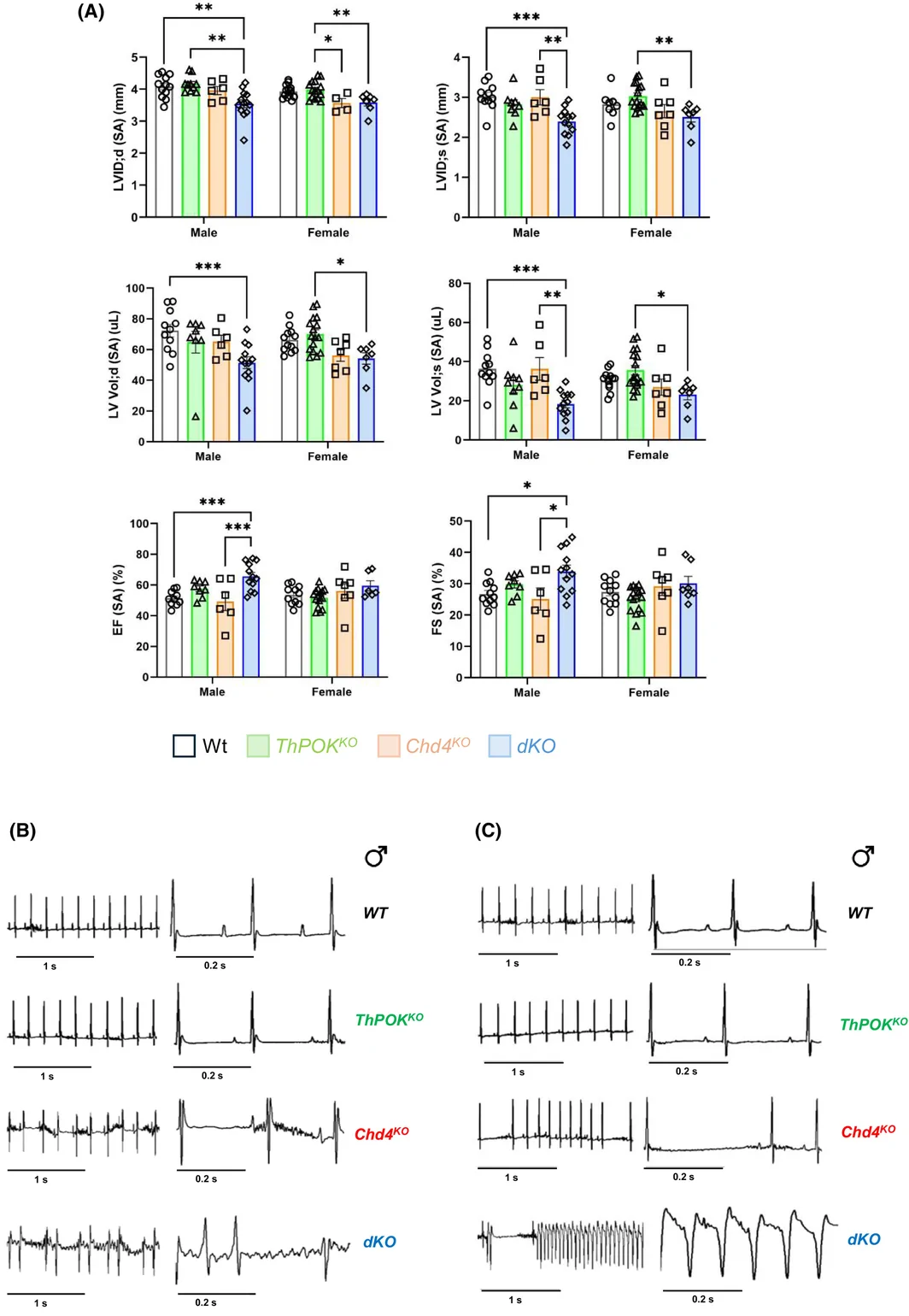
Echocardiographic and electrocardiographic analysis in WT and mutant mice. (A) At 10 weeks of age, *dKO* male and female mice exhibit pronounced cardiac remodeling compared to the other groups, characterized by significantly reduced left ventricular internal diameter in both diastole and systole, accompanied by decreased left ventricular volumes. Despite these reductions, *dKO* males show increased ejection fraction (EF) and fractional shortening (FS), consistent with their smaller ventricular size. Significance was determined using a two‐tail ANOVA (mean ± SEM). *P‐value < 0.05; **P‐value < 0.01; ***P‐value <0.001. (B) Electrocardiographic analysis of *WT*, *Chd4* ^*KO*^, *ThPOK* ^*KO*^ and *dKO* male mice at 5 weeks of age. Representative ECG traces from each group are shown. *dKO* mice exhibit severe arrhythmias, with atrial fibrillation being one of the most frequent phenotypes. (C) Electrocardiographic analysis of *WT*, *Chd4* ^*KO*^, *ThPOK* ^*KO*^ and *dKO* male mice at 10 weeks of age. Representative ECG traces from each group are shown. By 10 weeks of age, *dKO* mice develop severe arrhythmias, with atrial fibrillation and polymorphic non‐sustained ventricular fibrillation being the most common abnormalities.

At 5 weeks of age, *Chd4* ^*KO*^ and *dKO* females displayed significant arrhythmias, although no overt structural abnormalities were detected (Table S1). LV systolic performance was comparable among all groups at this stage, with no significant differences in LVID, EF, or LV volumes (Table S1). However, by 10 weeks of age, *dKO* females exhibited signs of systolic impairment, with significant decreases in LV mass and diastolic volume capacity, while systolic volumes remained largely unaffected (Fig. 3A and Table S1). These findings indicate that structural and functional abnormalities in *dKO* females emerge progressively with age.

In contrast, male *dKO* mice exhibited early cardiac remodeling already at 5 weeks of age, with a significant reduction in LVID in diastole compared to WT controls, accompanied by decreased LV volumes (Table S1). However, LV mass did not differ significantly among genotypes (Table S1). By 10 weeks of age, *dKO* males developed more pronounced systolic alterations, with marked reductions in LVID and LV volumes during both diastole and systole, compared not only to WT but also to *Chd4* ^*KO*^ and *ThPOK* ^*KO*^ mice (Fig. 3A). LV mass was significantly reduced in *dKO* animals as compared to all other groups, along with a further reduction in LV volumes during systole and diastole (Fig. 3A and Table S1). Interestingly, while *Chd4* ^*KO*^ mice maintained a preserved ejection fraction, *dKO* mice exhibited a marked increase in both ejection fraction and fractional shortening, consistent with their smaller heart size. Posterior wall thickness and right ventricular functional parameters remained unaffected across genotypes (Fig. 3A and Table S1).

To assess diastolic function, transmitral Doppler measurements were performed to calculate the E/A ratio, whereby a ratio of approximately 1.5 is considered normal. Both male and female *dKO* mice exhibited a significant increase in the E/A ratio at 5 weeks (not shown) and 10 weeks, with some animals displaying values exceeding 3.0 (Fig. S1A). Despite this marked increase, IVRT values did not differ significantly between *dKO* and WT mice, indicating that LV relaxation remained normal (Fig. S1A). These results suggested that both male and female *dKO* mice had diastolic dysfunction, likely indicative of restrictive left ventricular filling [22].

To further investigate the electrophysiological consequences of *Chd4* and *ThPOK* deletion, electrocardiograms (ECGs) were acquired from 5‐ and 10‐week‐old mice. At 5 weeks, both WT and *ThPOK* ^*KO*^ female mice exhibited normal electrocardiograms (ECGs) (Fig. S1B). As expected, *Chd4* ^*KO*^ females displayed arrhythmias, consistent with our previous findings [3]. The most common abnormalities in these animals included bigeminy and prolonged PR intervals, indicative of delayed atrioventricular conduction (Fig. S1B). This PR prolongation can, in fact, justify diastolic E/A ratio differences. In contrast, the concurrent deletions of *Chd4* and *ThPOK* (*dKO* mice) resulted in atrial fibrillation (AF), along with other electrical disturbances (Fig. S1B). AF was consistently observed in 5‐week‐old *dKO* females, though the severity and presentation of the arrhythmias varied among individuals, which is likely to reflect variability in phenotypic penetrance, as it is measured at an early timepoint.

By 10 weeks of age, ECG traces of WT, *ThPOK* ^*KO*^, and *Chd4* ^*KO*^ female mice seemed like those observed at 5 weeks. However, *Chd4* ^*KO*^ females showed a noticeable deterioration in their overall health, with an even more pronounced aggravation in the *dKO* group (Fig. S1C). At this later time point, AF and ventricular tachycardias (VT) were more frequently detected in both *Chd4* ^*KO*^ and *dKO* females, with the latter group displaying more severe electrophysiological abnormalities (Fig. S1C).

We also performed ECGs on male mice at 5 and 10 weeks of age. Like females, WT males exhibited normal ECG patterns at 5 weeks, and *ThPOK* ^*KO*^ males showed no arrhythmias (Fig. 3B). *Chd4* ^*KO*^ males predominantly displayed arrhythmias, including bigeminy and prolonged PR intervals, consistent with findings in females. Finally, *dKO* males exhibited AF and other electrical abnormalities (Fig. 3B).

At 10 weeks of age, ECG recordings from WT and *ThPOK* ^*KO*^ males remained unaltered, while *Chd4* ^*KO*^ males exhibited more pronounced arrhythmias, including AF and VT, but not ventricular fibrillation (Fig. 3C). In contrast, *dKO* males exhibited both AF and VT, with several animals also developing nonsustained polymorphic VT or ventricular fibrillation (Fig. 3C).

To further explore sex‐dependent differences in arrhythmia severity, we analyzed in more detail the proportion of animals that exhibited arrhythmias at 10 weeks of age, separated by sex (Fig. S1D). In contrast to the normal ECGs observed in all WT mice, a significant proportion of *ThPOK* ^*KO*^ females (13%) and males (8%) mice exhibited mild arrhythmias, primarily characterized by bradycardia and extrasystoles (Fig. S1D), which did not affect their survival throughout the period of study. In contrast to *ThPOK* mutants, significant differences were observed between *Chd4* ^*KO*^ and *dKO* male and female mice. Specifically, mild arrhythmias were detected in 80% of *Chd4* ^*KO*^ females, predominantly affecting the PR interval, which was prolonged in some cases (Fig. S1D). AF was observed in 13% of females, and 7% developed VT (Fig. S1D). *Chd4* ^*KO*^ males exhibited a higher incidence of AF (25%) and VT (8%). In the *dKO* group, electrophysiological abnormalities were even more pronounced, with marked sex‐dependent differences (Fig. S1D). Mild arrhythmias were present in 52% of *dKO* females as compared to 37% of males. Episodes of AF occurred in 38% of females and 47% of males. VT was twice as frequent in males (20%, 6 out of 30) as compared to females (10%, 3 out of 29). Furthermore, two *dKO* males experienced sudden death during ECG acquisition, likely due to ventricular fibrillation. These findings highlight the exacerbated impact of the *ThPOK* deletion on cardiac function when combined with *Chd4*, with a more severe electrical phenotype in male mice.

### Transcriptional analyses of *Chd4* and *dKO* mice reveal molecular determinants of arrhythmogenesis

To gain insight into the molecular mechanisms underlying the phenotypic abnormalities observed in *Chd4* and *Chd4;ThPOK*‐deficient mouse models, we performed RNA‐seq on whole‐heart samples from postnatal day 21 (P21). These analyses were performed at P21 to capture early transcriptional alterations preceding the onset of severe electrophysiological manifestations. Later stages of cardiac disease are often accompanied by broad activation of secondary stress and remodeling pathways that may obscure primary molecular events. By focusing on this early time‐point, we aimed to identify transcriptional programs more directly linked to *Chd4* and *ThPOK* loss. Differential gene expression analysis revealed a progressive transcriptional deregulation: while *ThPOK* ^*KO*^ hearts showed no significant changes, *Chd4* ^*KO*^ and *dKO* hearts exhibited upregulation of 703 and 935 genes (≥ 2‐fold), and downregulation of 224 and 601 genes (≤ 2‐fold), respectively (Table S2). This supports the lack of structural or histopathological abnormalities in *ThPOK* ^*KO*^ hearts and highlights the profound transcriptional impact of *Chd4* deletion, further amplified in the double mutants.

Comparative analyses of single *Chd4* ^*KO*^ (*sKO*) and *dKO* mutants using the Enrichr platform provided insights into functional disparities. Gene ontology (GO) analyses of biological processes revealed enrichment in genes related to striated muscle contraction, neuronal signaling, and Ras‐mediated calcium influx in both groups (Fig. S2A). For molecular functions, *Chd4* ^*KO*^ hearts exhibited dysregulation in ionotropic glutamate receptor activity and glutamate‐gated and voltage‐gated ion channels (Fig. S2B, top), whereas *dKO* mice displayed marked upregulation in voltage‐gated cation and sodium channels, including those implicated in cardiac action potential generation (Fig. S2B, bottom). Since sarcomeric proteins and ion channel function are essential for proper cardiac rhythm, aberrant expression of skeletal muscle genes in terminally differentiated cardiomyocytes—combined with altered voltage‐gated ion channel expression—may collectively contribute to the arrhythmic phenotypes observed. These findings underscore the necessity to further dissect the additional molecular players exacerbating arrhythmias in *dKO* models.

The transcriptome data revealed significant alterations in ligand‐ and voltage‐gated ion channel transcripts, crucial regulators of cardiac impulse propagation, primarily in *Chd4* ^*KO*^ and *dKO* hearts. To investigate the molecular pathways contributing to electrophysiological stability in the myocardium, we investigated some ion channel genes, whose expression is upregulated in the mutants (Fig. 4A, upper left). In the Na + channel category, *Scn10a* expression (validated by qPCR) was substantially increased in both *Chd4* ^*KO*^ and *dKO*, with no significant statistical differences between them (Fig. 4A). *Scn5a* was slightly upregulated exclusively in *Chd4* ^*KO*^ mutants. Moreover, *Atp1a3*, which encodes a Na+/K+ ATPase highly expressed in cardiac and neuronal tissues, was upregulated in both *Chd4* ^*KO*^ and *dKO* hearts, by 42‐fold and 38‐fold, respectively. The skeletal muscle ATPase Ca2+ transporting *Atp2a1* (SERCA1) showed similar increases in both mutants. The fast‐twitch skeletal muscle Ca2+ sensor *Casq1* was elevated by over 15‐fold, while the cardiac calcium channel Cav3.2 (*Cacna1h*), crucial for pacemaker function, was upregulated more than fivefold as compared to WT. qRT‐PCR validations confirmed these RNA‐seq results (Fig. 4A).

**Fig. 4:**
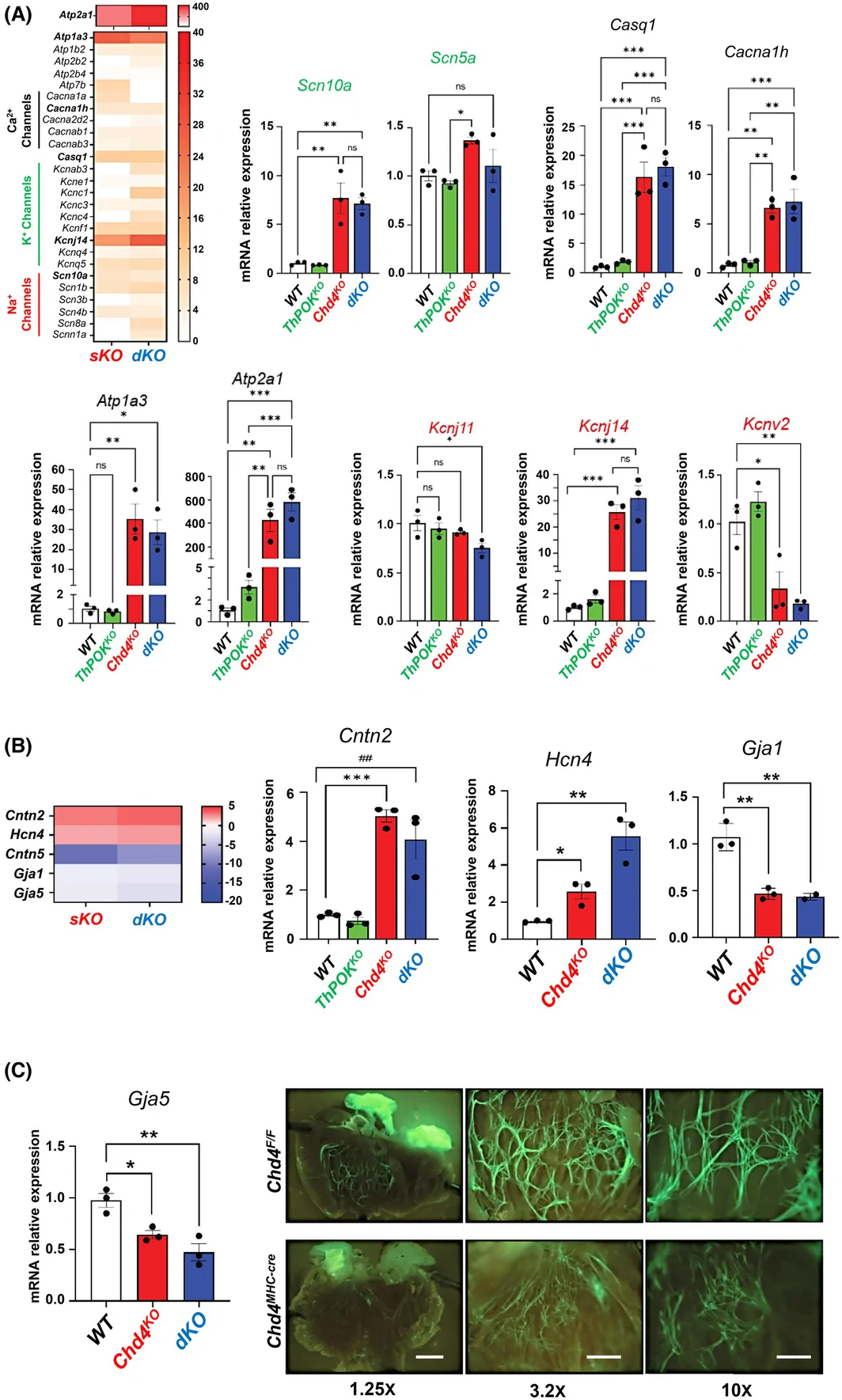
Transcriptomic analysis of WT, *ThPOK* ^*KO*^, *Chd4* ^*KO*^, *and dKO* mutant hearts. (A) Heatmap illustrating transcriptional changes in sodium, potassium, and calcium channel genes associated with action potential (AP) in *Chd4* ^*KO*^ (*sKO*) and *dKO* hearts relative to *WT* (upper left); Adjusted *P*‐value < 0.05. RT‐qPCR validation of selected AP genes from RNA‐seq data (right). (B) Heatmap showing deregulation of cardiac conduction system (*CCS*) genes in *sKO* and *dKO* hearts compared to *WT* (left). RT‐qPCR validation of *Cntn2* and *Hcn4* (upregulated) and *Gja1* (downregulated) transcripts (right). (C) RT‐qPCR validation of *Gja5* downregulation. Significance in A, B and C was determined using unpaired two‐sided t‐test (mean ± SEM). *P‐value < 0.05; **P‐value < 0.01, ##*P*‐value < 0.01 (*WT* vs. *dKO* in *Cntn2* plot); ***P‐value < 0.001. Representative fluorescence microscopy images (Cx40^eGFP^, right) of the cardiac conduction system in *Chd4* ^*f/f*^ (*WT* control group, upper panels) and *Chd4* ^*f/f*^ *;α‐MHC‐cre* mutant (*Chd4* ^*MHC‐Cre*^ mutant group, lower panels) (*n* = 3; 2 males and 1 female) are shown. Scale bars represent 2 mm (left), 500 μm (middle) and 200 μm (right image). The scale bars depicted in the mutant images are valid for the corresponding WT pictures. In each experimental group A and B, approximately 50% of the mice were male and 50% were female.

Potassium channel expression also differed among genotypes. *Kcnj14* was more upregulated in *dKO* than in *Chd4* ^*KO*^, though its expression differences between *Chd4* ^*KO*^ and *dKO* were not statistically significant. Conversely, *Kcnj11* was significantly downregulated in *dKO*, and *Kcnv2* showed comparable reduction in both mutants without significant differences between groups (Fig. 4A). These results support a role for Chd4/NuRD in repressing ion channel gene expression to maintain electrophysiological stability, with no marked contribution from ThPOK.

We next investigated the transcriptional regulation of cardiac conduction system (CCS) genes in *Chd4* and  *Chd4;ThPOK* mutant mice. RNA‐seq analysis revealed upregulation of contactin 2 (*Cntn2*) and hyperpolarization‐activated cyclic nucleotide‐gated potassium channel 4 (*Hcn4*), alongside downregulation of connexins 40 (*Gja5*) and 43 (*Gja1*) (Fig. 4B,C). RT‐qPCR confirmed these findings, with a not significant trend for greater *Cntn2* upregulation in *Chd4* ^*KO*^ than in *dKO*, a slight (but not significant) upregulation of *Hcn4* in *dKO* vs *Chd4* ^*KO*^, and similar downregulation of *Gja1* and *Gja5* in both mutants (Fig. 4B). Consistent with the reduced expression of *Gja5* detected in *Chd4*‐deficient hearts (Fig. 4C), we analyzed ventricular conduction system (VCS) morphology using the Cx40‐GFP reporter line and the *Myh6‐cre* mouse line (see the Materials and Methods section). In control hearts, GFP labeling revealed a well‐organized and intricate VCS network. In contrast, *Chd4* ^*KO*^ hearts displayed a markedly disorganized VCS with reduced GFP signal, indicating impaired development and structural disruption of the ventricular conduction system.

### Chd4 and ThPOK regulate transcriptional repression of the pro‐apoptotic gene *Sprr1a* in the heart: Implications of *microRNA‐150* and the nuclear lamina

To determine whether the combined deletion of *Chd4* and *ThPOK* induces additional transcriptional changes beyond those caused by *Chd4* loss alone, we performed a focused re‐analysis of our RNA‐seq datasets. Surprisingly, although *dKO* hearts exhibited a greater number of differentially expressed genes as compared to WT than did *Chd4* ^*KO*^ hearts, direct comparison between *dKO* and *Chd4* ^*KO*^ samples, followed by Ingenuity Pathway Analysis (IPA), identified only five transcripts as being significantly more upregulated in the *dKO* hearts: *Dcst1*, *Nppa* (*Anf*), *Rtl1*, *Sprr1a*, and *Sprr2f* (Fig. 5A and Table S2).

**Fig. 5:**
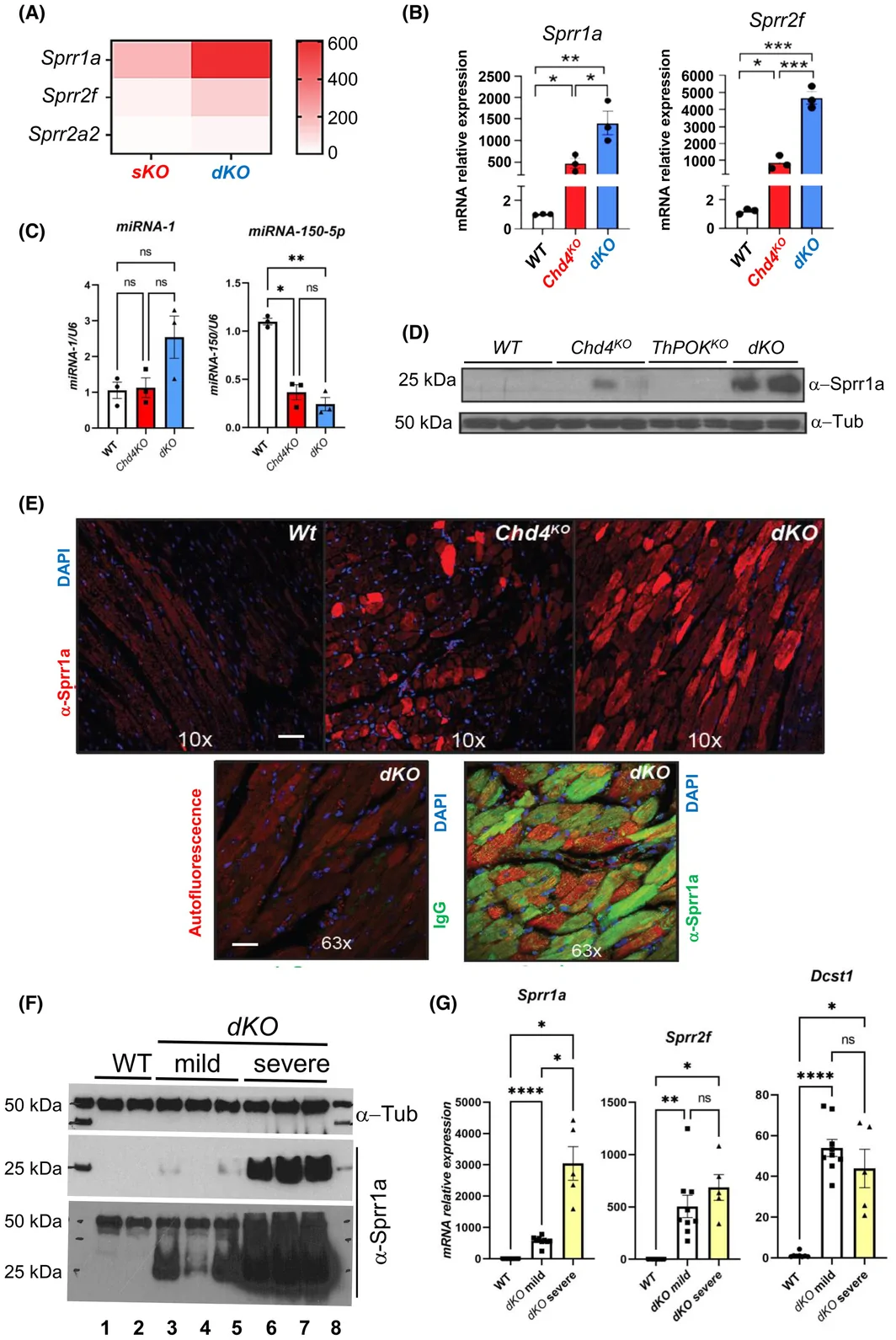
Deletion of *Chd4* and *ThPOK* prevents cardiac repression of Sprr1a. (A) RNA‐seq Heatmap showing upregulation of *Sprr* gene family members (*Sprr1a*, *Sprr2f* and *Sprr2a2*) in *Chd4* ^*KO*^ (*sKO*) *and dKO* hearts compared to WT. (B) RT‐qPCR validation of *Sprr1a* and *Sprr2f* upregulation in both mutant groups relative to WT (set around 1). (C) RT‐qPCR analysis of *miRNA‐1* and *miRNA‐150‐5p* expression in *Chd4* ^*KO*^ and *dKO* hearts compared to WT. (D) Western blot showing Sprr1a protein levels in representative WT, *Chd4* ^*KO*^, *ThPOK* ^*KO*^ and *dKO* mice. (E) Immunofluorescence images of WT, *Chd4* ^*KO*^ and *dKO* hearts showing Sprr1a expression in mutant cardiomyocytes (top). On the bottom, images from the same *dKO* heart illustrate tissue autofluorescence detected in the red channel (bottom left) and Sprr1a staining in green (bottom right), with red autofluorescence also visible. (F) Western blot analysis of Sprr1a protein heart levels in WT (lanes 1 and 2), *dKO* mice with mild ECG phenotype (lanes 3–5), and *dKO* with severe ECG phenotype (lanes 6–8). *Tubulin* (upper panel) serves as a loading control, and the lower panel shows an overexposed *Sprr1a* signal. (G) RT‐qPCR analysis of *Sprr1a*, *Sprr2f* and *Dcst1* expression in WT, *dKO* (mild phenotype) and *dKO* (severe phenotype) hearts. Significance in B and C was determined using unpaired two‐sided t‐test. Statistical analysis for G was performed using Brown–Forsythe and Welch ANOVA to compare the three groups (mean ± SEM). *P‐value < 0.05; **P‐value < 0.01; ***P‐value < 0.001; ****P‐value < 0.0001. White scale bars represent 200 μm and 50 μm for 10 × and 63 × magnification pictures, respectively. The scale bars depicted in the WT images are valid for the corresponding mutant pictures. The sexes of the mice were as indicated in Fig. 4A,B.

Further validation by RT‐qPCR revealed that while *Nppa* (*Anf*) showed only modest upregulation in *dKO* hearts, likely reflecting an enhanced stress response, *Dcst1*, *Rtl1*, *Sprr1a* and *Sprr2f* were all significantly elevated in *dKO* relative to *Chd4* ^*KO*^ hearts (Figs. 5B and S3A). While *Sprr1a* and *Sprr2f* were not identified as differentially expressed in *ThPOK* ^KO^ hearts by RNA‐seq, RT‐qPCR reanalysis revealed mild but significant upregulation of both genes in *ThPOK* ^KO^ versus WT hearts (Fig. S3B). Importantly, this partial derepression was not observed for the other transcripts more highly expressed in *dKO* hearts compared to *Chd4* ^*KO*^, whose expression levels in *ThPOK* ^KO^ hearts remained comparable to WT (Fig. S3A). These findings suggest a selective regulatory contribution of ThPOK to the control of *Sprr1a* and *Sprr2f* repression in the heart.

*Sprr1a* (also known as cornifin‐A) is a member of the small proline‐rich protein family, classically involved in keratinocyte cornification, and has been reported to exhibit ectopic expression in human cardiac tissue following myocardial infarction [17]. Since *Sprr1a* expression is post‐transcriptionally repressed by *microRNA‐150* [17], we evaluated *miRNA‐150‐5p* levels in our mutant hearts. Both *Chd4* ^*KO*^ and *dKO* hearts exhibited similarly reduced levels of *miRNA‐150‐5p* compared to WT, indicating that this downregulation is primarily driven by *Chd4* loss, with no additional effect from *ThPOK* deletion (Fig. 5C). As a negative control, we analyzed *miRNA‐1*, a cardiac‐enriched microRNA not implicated in *Sprr1a* regulation, and observed no differences in its expression across genotypes, supporting the specificity of the observed *miRNA‐150‐5p* changes.

At the protein level, western blot analysis confirmed an elevated Sprr1a expression in mutant hearts, with the highest levels observed in *dKO* animals (Fig. 5D). Immunohistochemical analysis localized Sprr1a protein predominantly in the cytoplasm of cardiomyocytes, further confirming the highest expression in *dKO* hearts (Fig. 5E). These results indicate that Chd4 and ThPOK cooperatively repressed *Sprr1a* expression and that their combined loss likely contributed to the aggravated cardiac phenotype observed in *dKO* mice.

To explore whether Sprr1a expression correlates with the severity of the electrical phenotype, we stratified *dKO* mice into cohorts exhibiting either mild or severe conduction defects at the time of sacrifice (Fig. S3C). Western blot analysis revealed significantly higher levels of Sprr1a protein in hearts from *dKO* mice with severe electrical abnormalities compared to those with milder phenotypes, supporting an association between Sprr1a overexpression and adverse cardiac outcomes (Fig. 5F). RT‐qPCR analysis confirmed that *Sprr1a* was the only gene among the candidates tested whose expression consistently increased in mice with severe phenotypes compared to milder cases (Fig. 5G), reinforcing its potential role as a marker or driver of disease progression.

In human‐induced pluripotent stem cell‐derived cardiomyocytes (hiPSC‐CMs), ThPOK localizes at the nuclear lamina, where it is involved in maintaining transcriptional repression through the anchorage of heterochromatic regions [14, 15]. Based on this, we hypothesized that the upregulation of *Sprr1a* in our mutant models could result from the loss of nuclear lamina tethering of the *Sprr1a* locus, leading to its transcriptional derepression. To address this, we examined hearts from mice carrying the *Lmna‐R249W* mutation, a pathogenic variant known to cause striated muscle laminopathy and cardiac dysfunction [23, 24]. Strikingly, expression analysis revealed strong upregulation of *Sprr1a*, *Sprr2f*, and a significant upregulation of *Dcst1* in heterozygous *Lmna* ^*R249W*^ mutant hearts (Fig. 6A and Fig. S3D), highlighting the critical role of nuclear lamina integrity in maintaining the transcriptional silencing of the *Sprr* gene family.

**Fig. 6:**
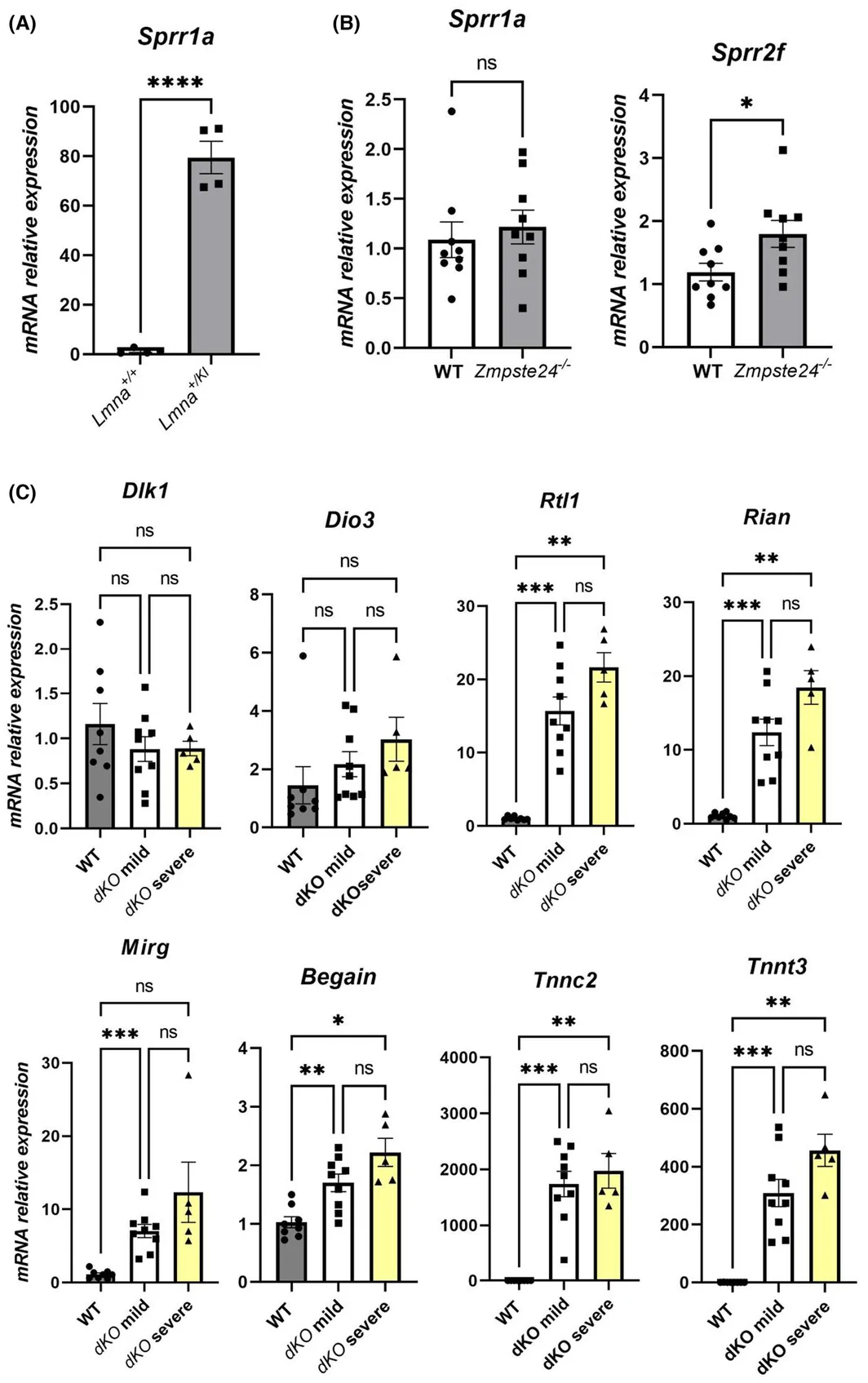
Transcriptional profiling of mouse laminopathy models and analysis of the *Dlk1*‐*Dio3* mega gene cluster in WT and *Chd4;ThPOK dKO* hearts with mild or severe electrical phenotypes. (A) RT‐qPCR analysis of *Sprr1a* mRNA expression in WT and *Lmna* ^*R249W*^ mutant hearts (*n* = 4 male per group). (B) RT‐qPCR of *Sprr1a* and *Sprr2f* expression in WT (*n* = 9; 5 male and 4 female) and *Zmpste24* ^*−/−*^ (*n* = 9; 5 male and 4 female) hearts. (C) RT‐qPCR analysis of selected transcripts from the *Dlk1‐Dio3* gene cluster in *dKO* hearts with mild or severe electrical phenotypes (as in Fig. 5F,G), compared to *WT. m36B4* was used as a reference gene, and expression values are presented relative to *WT* (set to 1). Significance in A and B was determined using unpaired two‐sided t‐test. Statistical analysis for C was performed using Brown–Forsythe and Welch ANOVA to compare the three groups (mean ± SEM). *P‐value < 0.05; **P‐value < 0.01; ***P‐value < 0.001; ****P‐value < 0.0001 and ns = not significant.

To further test the specificity of *Sprr1a* induction, we analyzed hearts from Zmpste24‐deficient mice, which lack the zinc metallopeptidase STE24 (ZMPSTE24) required for prelamin A maturation [25]. As a consequence of the accumulation of unprocessed prelamin A, prominent alterations in the nuclear envelope have been described in *Zmpste24* ^*−/−*^ mice [26]. However, in contrast to the *Lmna* ^*R249W*^ model, *Zmpste24* deficiency primarily causes a premature aging phenotype resembling Hutchinson‐Gilford progeria syndrome (HGPS), with comparatively mild and delayed cardiac involvement [27]. Analysis of heart tissue from *Zmpste24* ^*−/−*^ mice revealed no detectable ectopic expression of *Sprr1a* (Fig. 6B). These findings suggest that Sprr1a induction is not a general consequence of nuclear envelope defects but may be specifically associated with laminopathies that directly compromise cardiomyocyte nuclear integrity and lead to early and severe cardiac dysfunction.

### Regulation of *Dlk1*‐*Dio3* mega gene cluster repression by Chd4

The retrotransposon‐derived imprinted gene *Rtl1* is among the most highly upregulated genes in *dKO* compared to *Chd4* ^*KO*^ hearts. *Rtl1* is embedded within the *Dlk1‐Dio3* mega gene cluster, a large, imprinted locus essential for heart development [28]. This region encodes not only protein‐coding genes but also an array of long noncoding RNAs (*lncRNAs*) and microRNAs (*miRNAs*) that are critical regulators of cardiac morphogenesis and of mitochondrial protein synthesis—an essential process for glycolysis in skeletal muscle [29, 30, 31]. Given the robust upregulation of *Rtl1* in *Chd4* ^*KO*^ hearts and its further increase in *dKO* hearts, we investigated whether other genes within the *Dlk1‐Dio3* cluster followed a similar expression pattern.

RT‐qPCR analyses of representative cluster components—including the protein‐coding boundary genes *Dlk1* and *Dio3*, and the noncoding RNAs *Meg3 (Gtl2)*, *Rian*, and *Mirg*—revealed that *Dlk1* levels remained unchanged across genotypes, while *Dio3* expression was stable except in one *dKO* outlier with a ~ 30‐fold increase (Fig. S4). *Meg3* and *Rian* were significantly upregulated in both *Chd4* ^*KO*^ and *dKO* hearts, with no further increase in the *dKO*, indicating that their derepression is primarily due to Chd4 loss. *Mirg* showed a not significant trend toward higher expression in *dKO* hearts, again driven by the same outlier sample. We also assessed *Begain*, a gene immediately upstream of the cluster, which was similarly upregulated in both *Chd4* ^*KO*^ and *dKO* hearts. These results suggest that Chd4 broadly represses transcription across the *Dlk1*‐*Dio3* region and neighboring loci.

To assess whether differential expression of *Dlk1*‐*Dio3* cluster genes contributes to the severity of the electrical phenotype observed in *dKO* mice, we evaluated the transcriptional levels of *Dlk1*, *Rian*, and *Mirg* in *dKO* animals stratified by mild or severe conduction defects. Not significant differences were observed between these groups, indicating that the expression of these genes does not correlate with phenotype severity (Fig. 6C). In contrast, positive control genes such as the skeletal muscle genes *Tnnc2* and *Tnnt3*, previously shown to be upregulated in the Chd4 mutant model [3], displayed the expected increase in *dKO* hearts compared to WT, independent of disease severity.

Because the nuclear lamina helps organize chromatin domains, we examined whether its disruption contributes to *Dlk1‐Dio3* derepression. To address this, we analyzed hearts from mice carrying the *Lmna‐R249W* mutation, which disrupts nuclear lamina structure and causes cardiac laminopathy [30, 32]. Interestingly, none of the *Dlk1‐Dio3* cluster genes were upregulated in these mutants (Fig. S5A). Additionally, qPCR analyses of hearts from HGPS model *Zmpste24* ^*−/−*^ mice confirmed that either *Dcst1* or the *Dlk1‐Dio3* cluster embedded gene *Mirg* were not misexpressed, further supporting the notion that the repression of these genes is specifically dependent on Chd4, rather than on general disruptions of nuclear envelope integrity (Fig. S5B).

In summary, these results indicate that while nuclear lamina defects can selectively impact on the expression of certain genes such as *Dcst1*, the integrity of the *Dlk1‐Dio3* cluster repression appears to rely predominantly on Chd4 function. The severe electrical dysfunction and high incidence of sudden cardiac death observed in *dKO* mice likely resulted from a combination of Chd4‐ and ThPOK‐dependent mechanisms, with *Sprr1a* upregulation playing a putative pathological role. Altogether, this work highlights the complex crosstalk between chromatin remodeling and nuclear architecture in safeguarding cardiac transcriptional stability and preventing arrhythmogenic heart failure.

## Discussion

This study unveils a previously unrecognized epigenetic safeguard mechanism in the heart, whereby the chromatin remodeler Chd4/NuRD and the transcription factor ThPOK cooperate to repress maladaptive gene expression programs and to preserve the structural and electrophysiological integrity of the adult myocardium. While the roles of chromatin remodelers and nuclear lamina‐associated factors in cardiac development have been individually documented, our data point to a unique synergy between Chd4 and ThPOK that orchestrates chromatin architecture and nuclear positioning to silence stress‐responsive loci such as *Sprr1a*, thereby preventing arrhythmias and maladaptive remodeling (Fig. 7).

**Fig. 7:**
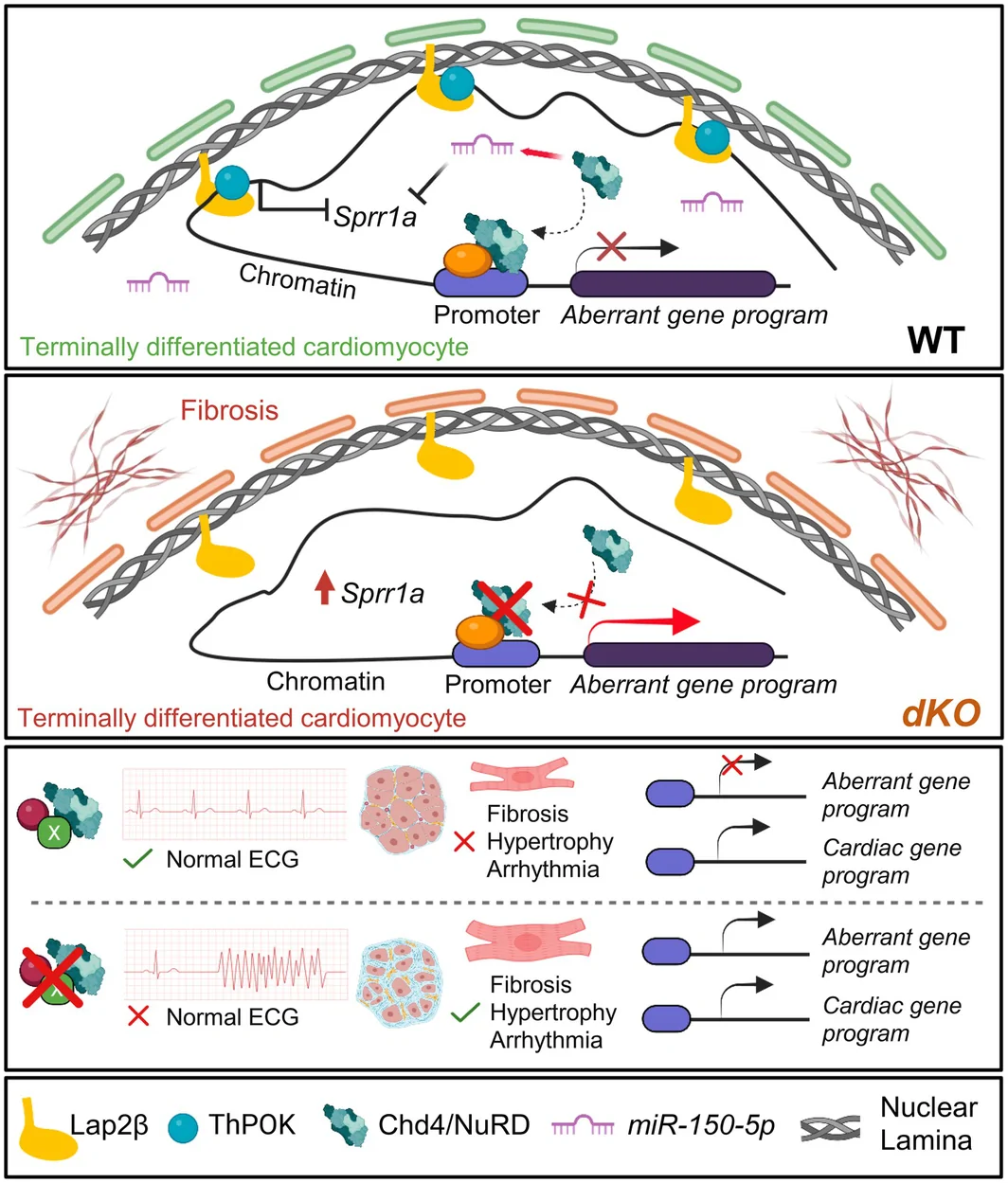
Model of the Chd4;ThPOK regulatory axis controlling Sprr1a repression in adult cardiomyocytes. Schematic model illustrating how Chd4/NuRD and ThPOK cooperate to maintain transcriptional repression and nuclear organization in terminally differentiated cardiomyocytes. In wild‐type (WT) hearts, Chd4‐dependent repression and *miR‐150‐5p* expression, together with ThPOK‐mediated chromatin organization at the nuclear lamina, contribute to silencing of the *Sprr1a* locus and maintenance of the cardiac gene program and normal electrical activity. In contrast, combined loss of *Chd4* and *ThPOK* (*dKO*) disrupts these regulatory mechanisms, leading to *Sprr1a* derepression, activation of aberrant gene programs, and the development of cardiac fibrosis, cardiomyocyte hypertrophy, and electrophysiological abnormalities. Figure made with the Instituto de Salud Carlos III institutional license of BioRender.

Although ThPOK is traditionally associated with T‐cell lineage commitment, our work uncovers its function in a completely different context: postnatal cardiomyocytes, where it remains largely dispensable under basal conditions, possibly due to compensation by related Zbtb family members, such as Pokemon (Zbtb7a), which share DNA‐binding sequences and regulatory functions [16, 33]. However, in the absence of Chd4, ThPOK becomes essential for restraining abnormal gene expression, revealing a latent functional redundancy that is only uncovered upon epigenetic stress. This interdependence becomes evident in the *Chd4;ThPOK* double knockout (*dKO*) model, which exhibits profound electrical abnormalities, structural disorganization, and defective cardiac conduction (Fig. 7).

The transcriptomic alterations in *Chd4* ^*KO*^ and *dKO* hearts delineate a molecular framework for the observed electrophysiological defects. Among the changes identified by RNA‐seq, several transcriptional alterations most directly support the central regulatory model proposed here, including the synergistic induction of Sprr1a in *dKO* hearts, the reduction of *miR‐150‐5p* upon *Chd4* ablation, and the coordinated deregulation of genes involved in cardiac conduction and electrical coupling. Upregulation of pacemaker‐related genes such as *Hcn4* and *Cntn2*, together with the downregulation of key gap junction proteins *Gja5 (Cx40)* and *Gja1 (Cx43)*, suggests a mechanistic basis for the arrhythmic phenotype observed in mutant hearts. Elevated Hcn4 expression may increase funny current (If), predisposing to ectopic impulse generation and arrhythmicity in failing hearts [34]. Simultaneously, reduced levels of Cx40 and Cx43 impair electrical coupling, facilitating reentrant arrhythmias [35]. While conduction defects were observed in both *Chd4* ^*KO*^ and *dKO* hearts, these changes are exacerbated in *dKO*s, implying that ThPOK contributes to the fine‐tuning of electrical homeostasis, perhaps through lamina‐associated gene silencing.

A closer look at the ventricular conduction system (VCS) revealed underdevelopment in *Chd4* mutants, consistent with reduced *Cx40* expression and conduction delays. Using the *Cx40* ^*eGFP*^ reporter line [36] in a *Myh6‐Cre* background, we circumvented embryonic lethality associated with early *Chd4* deletion [3], documenting underdevelopment and aplasia of the VCS. Although technical limitations precluded similar analysis in *dKO*s due to chromosomal proximity between *Zbtb7b* and *Cx40*, our findings point to a critical role for Chd4 in VCS maturation and raise the possibility that ThPOK also participates in this process by anchoring key loci to repressive nuclear compartments.

Sex‐specific cardiac remodeling in *dKO* mice further highlights the complexity of this regulatory axis. Male *dKO* mice showed reduced left ventricular mass, thinner walls, and smaller chamber volumes, accompanied by increased ejection fraction and fractional shortening, which is suggestive of a compensatory response to reduced preload and increased myocardial stiffness [37]. In contrast, female *dKO* mice developed diastolic dysfunction without overt systolic compromise, resembling heart failure with preserved ejection fraction (HFpEF), albeit without classical concentric hypertrophy or pulmonary edema. The heightened susceptibility observed in male mice may be influenced by sex‐dependent differences in hormonal signaling and chromatin architecture, both of which are known to modulate cardiac transcriptional programs and could interact with chromatin remodeling pathways. These findings point to potential hormonal or molecular differences in the cardiac response to stress and warrant further investigation.

*Sprr1a* emerged in this study as a prominent transcriptional target associated with the pathological phenotype resulting from *Chd4* deficiency, with its expression further enhanced in an additive manner in *dKO* hearts. Although traditionally recognized for its role in keratinocyte cornification, Sprr1a has recently been implicated in cardiac pathology, including postinfarction remodeling, with studies reporting a transcriptional upregulation of approximately fivefold in the hearts of heart failure (HF) patients [17]. Consistent with these findings, other members of the *Sprr* gene family, such as *Sprr2a* and *Sprr2b*, are similarly upregulated in infarcted rat hearts [38]. Additional *Sprr* family members, including *Sprr2b* and *Sprr3*, are induced in cardiac fibroblasts during HF, where their expression has been linked to pathological processes such as fibrosis, myocardial hypertrophy, and increased ventricular stiffness [39, 40]. In our models, however, Sprr1a is ectopically expressed in cardiomyocytes, and its expression levels correlate with the severity of arrhythmic phenotypes. Although Sprr1a induction represents one of the most robust molecular changes associated with disease severity in Chd4 and Chd4/ThPOK mutant hearts, our data do not establish a direct causal role for Sprr1a in driving the arrhythmogenic phenotype. Given the widespread transcriptional and metabolic alterations observed in these models, Sprr1a upregulation may therefore reflect broader chromatin and nuclear architectural disruption rather than acting as an isolated mechanistic driver. Future gain‐ and loss‐of‐function studies will be required to determine whether Sprr1a directly contributes to electrical instability or instead serves primarily as a molecular indicator of severe transcriptional deregulation.

Mechanistically, we propose two levels of regulation for *Sprr1a* derepression in *Chd4;ThPOK dKO* hearts. First, Chd4 loss leads to reduced expression of *miRNA‐150‐5p*, a known post‐transcriptional repressor of *Sprr1a*, likely contributing to its increased mRNA stability and protein expression. This is consistent with reports of *miRNA‐150‐5p* downregulation in cardiovascular disease and heart failure models [17]. In contrast, *ThPOK*‐deficient hearts display only a modest increase in Sprr1a transcript levels, and preserved miR‐150 expression likely restrains the accumulation of Sprr1a protein. This observation suggests that ThPOK alone is not a dominant regulator of adult cardiomyocyte homeostasis but rather exerts a context‐dependent modulatory role that becomes functionally relevant when Chd4‐dependent repression is compromised. Second, the additional loss of ThPOK, which is implicated in the formation of lamina‐associated domains (LADs), likely disrupts nuclear lamina‐mediated repression at the *Sprr1a* locus (Fig. 7) [41]. The gene harbors a conserved (GAGA)_n_‐rich sequence, a known DNA‐binding motif for ThPOK (originally identified in *Drosophila* as GAGA‐associated factor, or GAF [42]), suggesting that ThPOK anchors the locus to the nuclear periphery to maintain transcriptional silencing in cooperation with Chd4/NuRD. Disruption of this complex balance, either through *ThPOK* loss, lamina disorganization (as seen in laminopathies), or *Chd4* depletion, may cause relocalization of *Sprr1a* to more euchromatic, transcriptionally permissive regions. Together, these observations support the existence of two complementary mechanisms—one dependent on Chd4‐mediated miRNA regulation and another linked to ThPOK and nuclear lamina function—that converge on Sprr1a repression in cardiomyocytes.

An additional layer of transcriptional dysregulation uncovered in our study involves the imprinted *Dlk1*–*Dio3* cluster, whose activation appears to be specifically driven by the loss of Chd4 and does not exhibit dependence on ThPOK or on nuclear lamina integrity. Multiple components of this locus, including the lncRNAs *Meg3*, *Rian*, and *Mirg*, as well as *Rtl1*, were markedly upregulated in *Chd4*‐deficient hearts without further elevation in *dKO* cardiomyocytes, and remained unchanged in *Lmna* mutant models. This suggests that repression of the *Dlk1–Dio3* cluster relies on a Chd4/NuRD‐dependent epigenetic mechanism that is distinct from lamina‐associated gene silencing. Given that dysregulation of this locus has been implicated in cardiac morphogenesis and in mitochondrial protein synthesis [29, 30, 31], both processes previously linked to the cardiac phenotype of *Chd4* deficiency [3], its broad upregulation likely contributes to the overall pathological remodeling observed in our models. Although not predictive of phenotype severity, activation of the *Dlk1–Dio3* cluster may represent a foundational component of the transcriptional reprogramming triggered by loss of Chd4 in the adult heart, acting in parallel to lamina‐sensitive pathways such as those influencing Sprr1a.

Our model aligns with observations in mouse models of laminopathies, in which mutations in nuclear lamins such as *Lmna* ^*R249W*^ disrupt chromatin–lamina interactions, leading to upregulation of *Sprr1a* and related genes. We speculate that this mutation may recapitulate the loss of ThPOK function in anchoring the *Sprr1a* locus to the nuclear lamina, reinforcing the idea that ThPOK serves as a critical adaptor between GAGA‐rich genomic domains and the nuclear periphery. Remarkably, the vertebrate ortholog of ThPOK, vGAF, is expressed in the zebrafish heart and regulates developmental gene programs including *Hox*, supporting a conserved role in chromatin regulation [43].

The derepression of *Sprr1a* may also reflect a broader failure in establishing a repressive chromatin landscape. NuRD complexes contain histone 3 (H3) deacetylase enzymes HDAC1 and 2, and their absence likely impairs deacetylation‐dependent recruitment of methyltransferase activities associated with H3K9me2 or H3K27me3 deposition. Studies from the Reddy laboratory have shown that the chromosomal region containing the *Sprr* gene cluster resides within lamina‐associated domains (LADs) in several noncardiomyocyte cell types and loses lamina association upon combined inhibition of H3K27me3 and H3K9me2 [44]. This transition from acetylated to methylated chromatin is crucial for anchoring gene loci to the lamina [45]. Although lamina tethering of the *Sprr1a* locus has not been directly demonstrated in adult cardiomyocytes, these findings raise the possibility that Chd4/HDAC‐dependent chromatin transitions may contribute to maintaining lamina‐associated repression of the *Sprr* cluster. In this context, loss of Chd4—and potentially ThPOK‐mediated lamina organization—could render the *Sprr1a* locus both spatially and epigenetically derepressed.

Collectively, our findings uncover a novel regulatory axis involving Chd4/NuRD and ThPOK that safeguards cardiac homeostasis by repressing maladaptive transcriptional programs and supporting conduction system integrity. Disruption of this axis leads to severe arrhythmias, structural remodeling, and diastolic dysfunction through a combination of transcriptomic dysregulation, impaired chromatin‐lamina tethering, and ectopic activation of stress‐responsive loci such as *Sprr1a*. These data provide insight into how chromatin and nuclear architecture interact to maintain cardiomyocyte identity and function and suggest potential therapeutic avenues targeting this axis for the treatment of cardiac arrhythmias and fibrosis, particularly in diseases such as laminopathies involving lamina dysfunction or epigenetic deregulation.

## Materials and methods

### Animal models and genotyping

The *Chd4* ^*floxed*^ transgenic mouse strain has been previously described [21]. The mouse line *Zbtb7b* (*ThPOK*)^*floxed*^ was kindly provided by Dr. Daniel Mucida (The Rockefeller University), with permission of Dr. Dan R. Littman (New York University), in whose laboratory it was originally generated [19]. The *Cx40* ^*KIeGFP*^ transgenic line was generously provided by Dr. Lucile Miquerol [36]. The transgenic Cre mouse strains used were *α‐MHC* ^*Cre/+*^ (*Myh6* ^*Cre/+*^) and *Mck* ^*Cre/+*^ [20, 46]. *Zmpste24* ^*−/−*^ mouse model was kindly provided by Dr. Carlos López‐Otín (Universidad de Oviedo) [26].

Animals were maintained under standard specific pathogen‐free conditions in a controlled environment with a 12‐h light/12‐h dark cycle, with standard chow diet and water available *ad libitum*. Mice were housed in ventilated cages with a maximum of five animals per cage and provided with environmental enrichment.

Details regarding the sex and age of animals used in each experiment are specified in the corresponding Results sections and figure legends.

All efforts were made to minimize animal suffering. For experiments requiring scheduled endpoint analysis (e.g., analyses at 5 or 10 weeks of age), animals were monitored regularly and only a small proportion showed overt signs of distress at those ages, as sudden death more commonly occurred at later stages. In survival studies, mice were maintained in social groups whenever possible and environmental enrichment was provided to reduce stress associated with long‐term housing. No analgesic or pharmacological interventions were administered, as these could interfere with the cardiac physiological and pathological parameters under investigation. Animal experiments were designed, conducted, and reported in accordance with the ARRIVE 2.0 guidelines, including the Essential 10 recommendations.

Animal studies were previously approved by the Centro Nacional de Investigaciones Cardiovasculares (CNIC) ethics committee, as well as licensed by authorities of the Madrid region under the PROEX 346/15. All animal procedures were performed in conformity with the EU Directive 2010/63EU and Recommendation 2007/526/EC, enforced in Spanish law under Real Decreto 53/2013. When the sacrifice was necessary, mice were humanely euthanized by cervical dislocation in accordance with institutional and ethical guidelines.

### Histological analysis

Hearts were fixed overnight with 4% paraformaldehyde (vol/vol) in PBS, followed by paraffin embedding and microtome sectioning (5 μm) for hematoxylin and eosin (H&E) staining using standard procedures. Immunohistochemistry (IHC) and immunofluorescence (IF) were conducted using standard procedures.

To analyze the morphology of the cardiac conduction system, adult *Chd4* ^*floxed*^/*Cx40* ^*KIeGFP*^ (WT) and *Chd4* ^*floxed*^/*MHC* ^*cre*^/*Cx40* ^*KIeGFP*^ *(Chd4* ^*MHC‐cre*^ *)* mice were euthanized at 4–5 weeks of age. Hearts were gently perfused with room temperature 1 × PBS and dissected *in situ* for further analysis. A longitudinal incision was made along the interventricular septum to open the heart, carefully preserving the integrity of the Purkinje fiber network. Each heart was examined individually under a stereomicroscope equipped for GFP fluorescence detection. Hearts were then fixed in 2% paraformaldehyde (PFA) overnight at 4 °C, followed by five 1‐h washes in 1 × PBS. Tissues were mounted using fluoromount‐G (Thermo Fisher Scientific, 00–4958‐02), and images were acquired using the Zeiss LMS700 confocal microscopy [36].

### Echocardiography and electrocardiography (ECG)

Ultrahigh resolution echocardiography was performed by blind operator with a linear 30‐MHz transducer (Vevo 2100, VisualSonics Inc.). Mice were lightly anesthetized with 1 to 1.5% isoflurane in 100% oxygen and placed on a heated table to preserve physiological body temperature. HR was monitored and anesthesia delivery was adjusted to maintain an HR of 450 ± 50 beats/min. Two‐dimensional and M‐mode (MM) was performed in parasternal long‐ and short axis views. LV ejection fraction EF was assessed by Teichholz formula using MM images displayed over time and obtained by a single line in the middle of LV. LV mass (mg) was calculated by the following formula LVmass = ((1.05 × ((LVID;d + LVPW + interventricular septum)^3^ −LVID;d^3^)) × 0.8) from diastolic LV MM images. To assess LV diastolic function, spectral Doppler was recorded for analyzing early (E wave) and late (A wave) velocities of mitral flow. Pulse‐wave Doppler of pulmonary outflow was recorded in the parasternal view at the level of the aortic valve in short axis view proximal to the pulmonary valve leaflets and aligned to maximize laminar flow. The velocity‐time integral was obtained by tracing the outer edge of the pulmonary outlet of the Doppler profile. TAPSE was measured by MM at the tricuspid lateral annulus in the apical four‐chamber view. Finally, the presence or absence of arrhythmias was assessed according to the ECG signal.

ECG tracings were recorded from sedated animals, as detailed elsewhere [47]. Briefly, ECG recordings were acquired for 60 s at 2 KHz sweep‐speed using a MP36R data acquisition workstation (Biopac Systems, Goleta, CA, USA).

### RNA sequencing

For transcriptional profiling of adult organs, whole hearts (including atria) were surgically isolated from 21‐day‐old WT or *ThPOK* ^*KO*^, *Chd4* ^*KO*^ or *Chd4;ThPOK dKO* mice. The isolated organs were individually submerged into 1 mL TRIsure buffer (Bioline, London, UK), immediately snap‐frozen in liquid N_2_, and stored at −80 °C. Tissue homogenization was performed using the MagNA lyser system (Roche, Basel, Switzerland), and total RNA was isolated. RNA from 5–6 hearts per genotype was pooled and purified on Qiagen‐RNA‐clean columns (Qiagen, Venlo, The Netherlands). Experiments were conducted in triplicate (3 WT and 3 mutant pools). RNA sequencing procedures were previously described [3].

### Statistical analysis

The GraphPad Prism software 9.0 was used for data analysis. Appropriate tests were chosen according to the data distribution, based on the Saphiro–Wilk normality test. Numerical data are presented as mean ± SEM. Differences between WT and mutant groups were statistically assessed using unpaired Student's *t*‐test for most experiments, as indicated in figure legends. The statistical significance of the incidence of different arrhythmias between the different mice groups was assessed using *Chi‐square* analysis. Statistical survival differences between WT and mutant mice were assessed using the Log‐rank (Mantel‐Cox) test in Kaplan–Meier survival curves. Differences between WT, *dKO* mild, and *dKO* severe were assessed using Brown–Forsythe and Welch ANOVA. Significance was considered at a *P*‐value lower than 0.05.

RT‐qPCR analysis. Quantitative real‐time PCR (RT‐qPCR) of genes of interest and housekeeping genes were performed on the same RNA‐pooled samples used for RNA‐seq experiments. SYBR Green PCR master mix (Applied Bio‐Systems) was used for qPCR analysis.

For miRNA, pooled RNAs were Reverse transcribed using the EXIQON RT microRNA PCR miRCURY LNA Kit (Qiagen, Germantown, MD, USA). *miRNA‐150‐5p* and *miRNA1* were spotted on custom array plates. The *U6* housekeeping gene was used as internal control. Assays were run in a C1000 Touch Thermal Cycler (Bio‐Rad, Madrid, Spain).

## Author contributions

F.A‐S. was involved in conceptualization, investigation, methodology, formal analysis, writing original draft, writing – review and editing. A.N. was involved in conceptualization, formal analysis, investigation, supervision, writing original draft, writing – review and editing. R.B. was involved in investigation, methodology, writing – review and editing. M.Y. was involved in investigation, methodology, writing – review and editing. L.C. was involved in conceptualization, formal analysis, writing – review and editing. G.P‐S. was involved in conceptualization, formal analysis, writing – review and editing. M.D. was involved in conceptualization, formal analysis, writing – review and editing. I.H. was involved in conceptualization, methodology, formal analysis, writing – review and editing. A.L.A. was involved in conceptualization, formal analysis, writing – review and editing. I.P.C. was involved in conceptualization, funding‐acquisition, methodology, formal analysis, writing – review and editing. A.R.F. was involved in conceptualization, funding‐acquisition, investigation, methodology, formal analysis, writing – review and editing. J.M.R. was involved in conceptualization, formal analysis, funding‐acquisition, writing – review and editing. P.G.A. was involved in conceptualization, formal analysis, funding‐acquisition, project administration, resources, investigation, validation, supervision, visualization, writing original draft, writing – review and editing.

## Conflicts of interest

The authors declare no conflict of interest.

## Supporting information


**Fig. S1.** Diastolic and Electrical dysfunctions in mutant mice.
**Fig. S2.** Gene Ontology analysis of deregulated pathways in mutant hearts.
**Fig. S3.** Transcriptional analysis in WT, *ThPOK* ^*KO*^, *Chd4* ^*KO*^ and *dKO* and in *Lmna* ^*R249W*^ mutant hearts and validation with electrical phenotypes.
**Fig. S4.** Transcriptional analysis of the *Dlk1*‐*Dio3* mega‐cluster in mutant hearts.
**Fig. S5.** Expression analysis of *Dlk1‐Dio3* cluster and related genes in nuclear lamina mutant hearts.

**Table S1.** Echocardiographic parameters in WT, *ThPOK* ^*KO*^, *Chd4* ^*KO*^, and *dKO* mice.

**Table S2.** Differential gene expression from bulk RNA‐seq analysis.

## Data Availability

All data generated or analyzed during this study are included in this published article and its supplementary information files. Differentially expressed genes between WT and mutant hearts are all provided in Supplementary Table S2.
