# Supplementary material for: Chd4 and ThPOK cooperate to preserve structural and electrophysiological integrity of the adult heart through Sprr1a repression
Source: FEBS J. 2026 May 15;293(21):6444–66. doi: 10.1111/febs.70583 (PMC13731919; doi:10.1111/febs.70583)
Supplement: Supplementary file 1 [file FEBS-293-6444-s002.pdf]

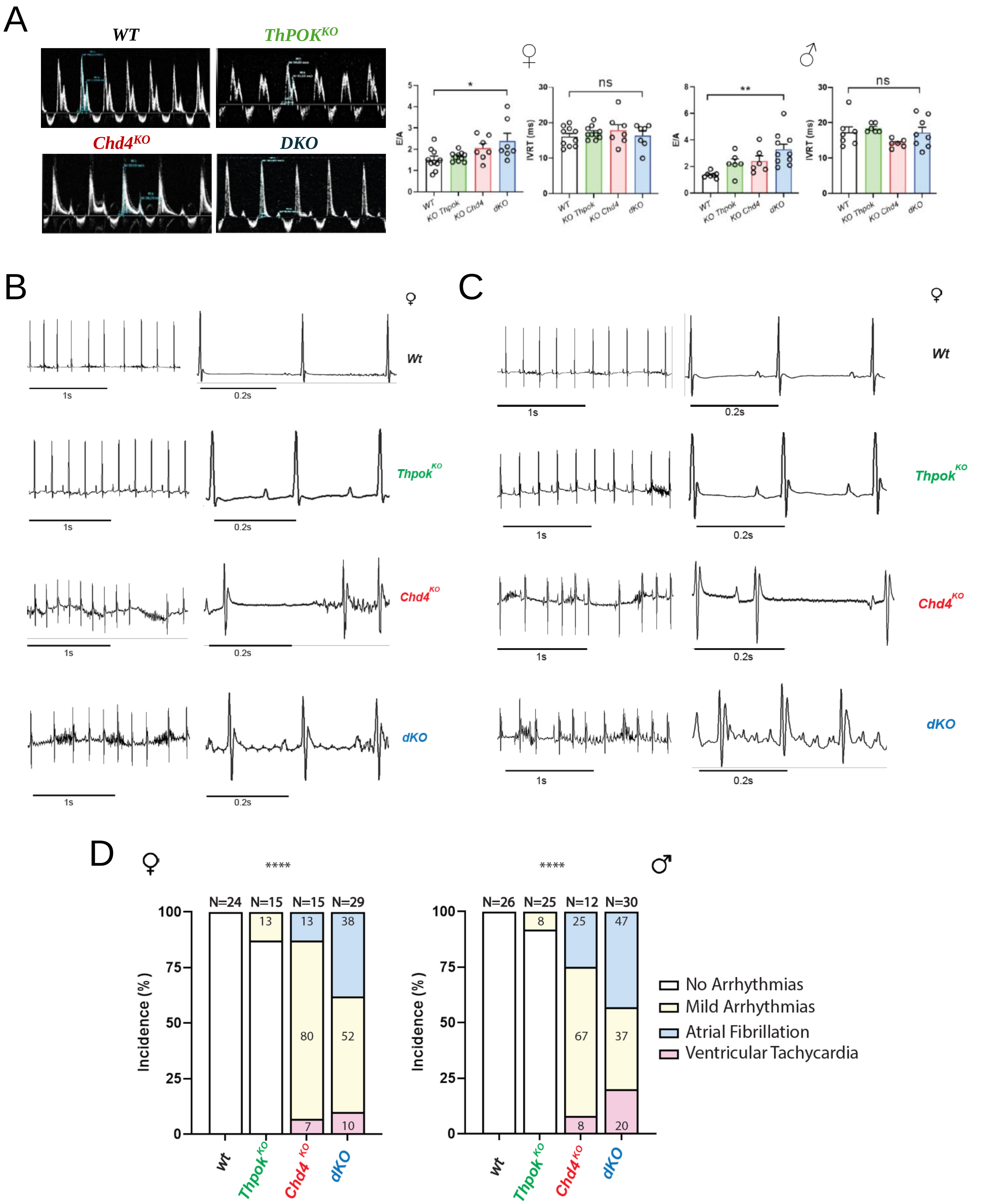

Figure S1. Diastolic and Electrical dysfunctions in mutant mice. **A,** Representative echocardiographic recordings at 10 weeks showing E and A wave measurements in *WT*, *ThPOK*^{KO}, *Chd4*^{KO}, and *dKO* groups (left). Quantification shows increased E/A ratios in both female and male *dKO* mice, with not significant alterations in isovolumetric relaxation time (IVRT) (right). Significance was determined using unpaired two-sided t-test (mean ± SEM). *p-value < 0.05; **p-value < 0.01; ns= not significant. **B**, Representative ECG traces of 5-week-old female *Wt* (n=20), *Chd4*^{KO} (n=15), *ThPOK*^{KO} (n=20) and *dKO* (n=20) mice. **C,** ECG traces of 10-week-old females from the same groups as in (B), showing increased incidence and severity of arrhythmias in *dKO* mice, including atrial fibrillation. **D,** incidence of arrhythmias in female (left) and male (right) *Wt*, *ThPOK*^{KO}, *Chd4*^{KO} and *dKO* mice. *ThPOK*^{KO} animals primarily exhibited mild bradyarrhythmias, while *Chd4*^{KO} and *dKO* males showed higher prevalence of ventricular tachyarrhythmias and atrial fibrillation. *dKO* mice exhibited the most severe phenotypes across sexes. Significance was determined using a *Chi-square* test. ****p-value< 0.0001.

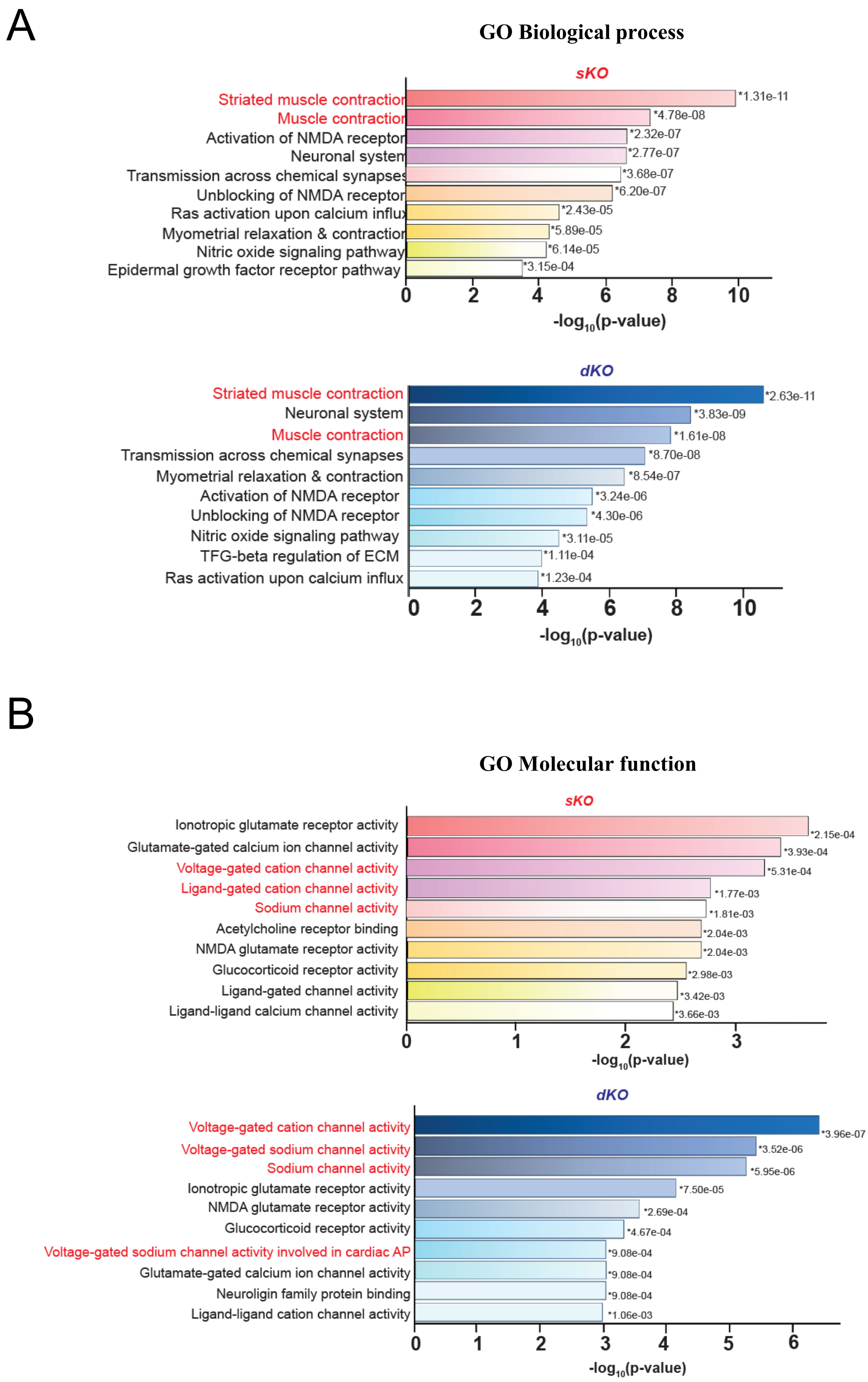

Figure S2. Gene Ontology analysis of deregulated pathways in mutant hearts. **A**, Enrichr analysis of significantly enriched biological processes in RNA-seq datasets from *Chd4^KO^* (*sKO*; upper) and *dKO* (lower) hearts compared to WT. **B**, Enrichr analysis of enriched molecular function terms in *Chd4^KO^* (upper) and *dKO* mice (lower). Data are expressed as $-\log_{10}$ (p-value).

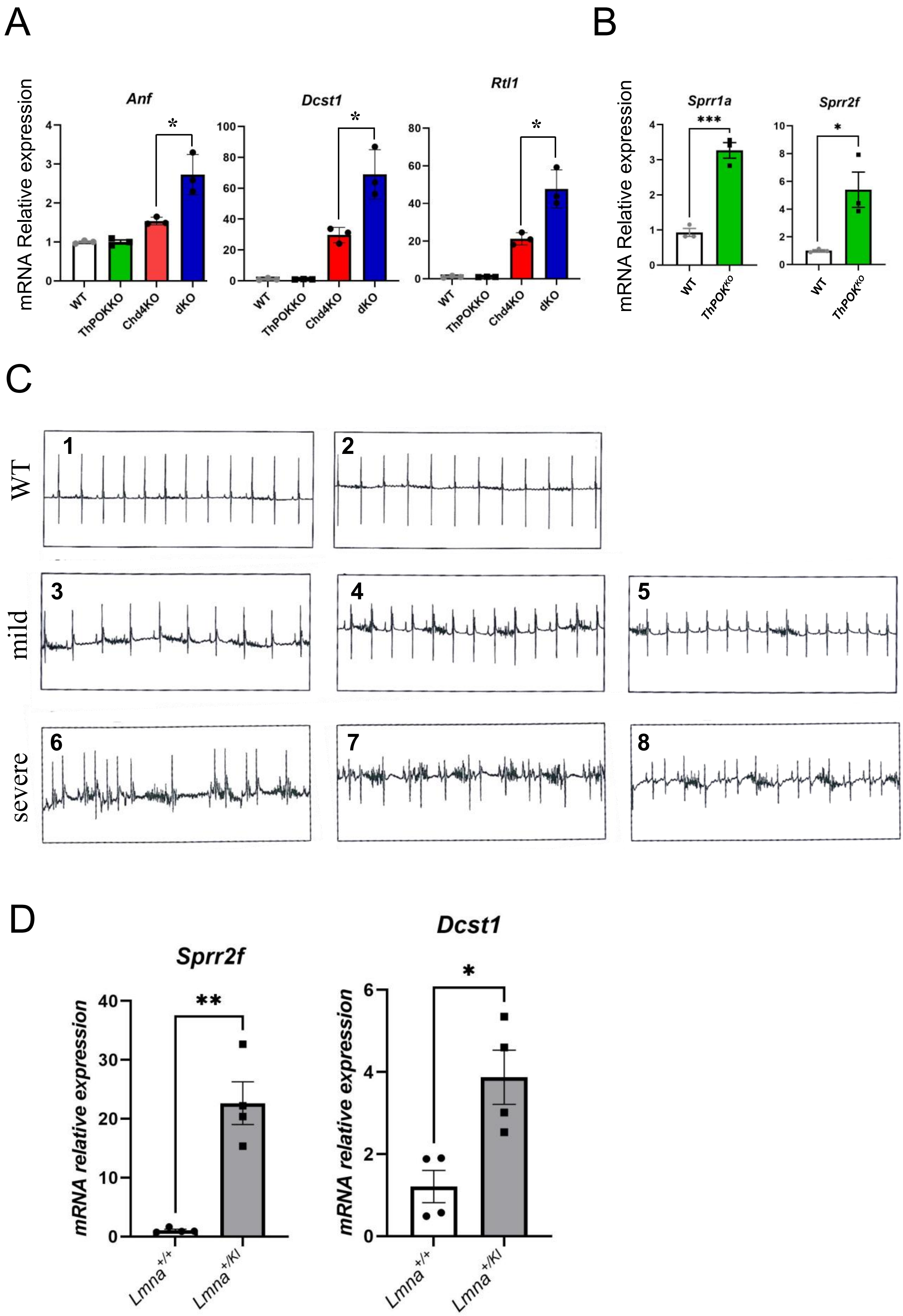

Figure S3. Transcriptional analysis in WT, *ThPOK*^{KO}, *Chd4*^{KO} and *dKO* and in *Lmna*^{R249W} mutant hearts and validation with electrical phenotypes. **A**, RT-qPCR validation of genes more upregulated in *dKO* than in *Chd4*^{KO} hearts. **B**, Expression of *Sprr1a* and *Sprr2f* in *ThPOK*^{KO} versus WT hearts. **C**, representative ECG recordings of *dKO* mice stratified by electrical phenotype severity, corresponding to data in Figure 5F. **D**, expression of *Sprr2f* and *Dcst1* in *Lmna*^{R249W} mutant and WT hearts by RT-qPCR (refer to Fig. 6A). Significance was determined using unpaired two-sided t-test (mean ± SEM). *p-value < 0.05; **p-value < 0.01; ***p-value < 0.001.

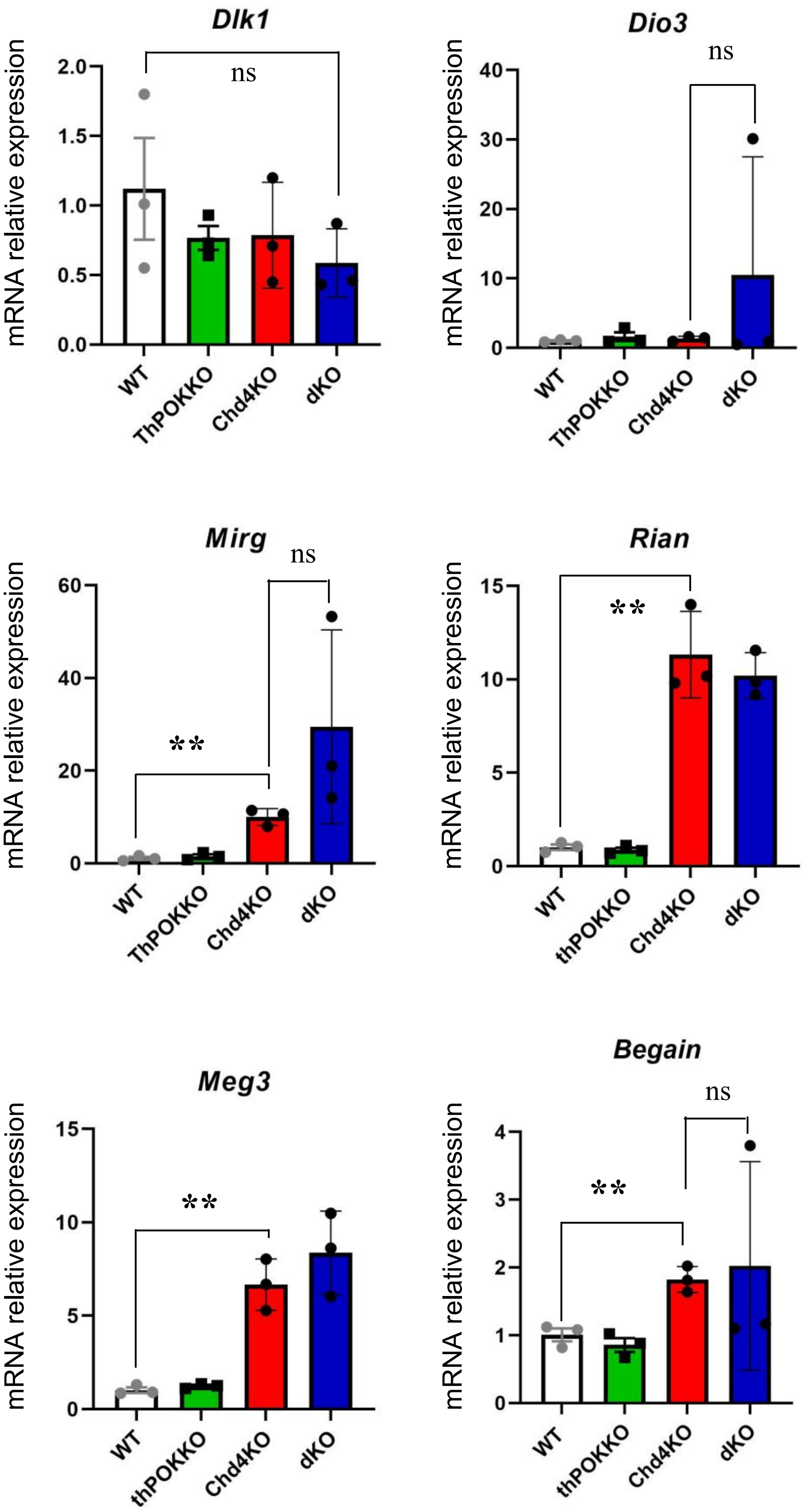

Figure S4. Transcriptional analysis of the *Dlk1-Dio3* mega-cluster in mutant hearts. RT-qPCR analysis of representative transcripts from the *Dlk1-Dio3* gene cluster in *WT*, *ThPOK*^{KO}, *Chd4*^{KO} and *dKO* hearts. Significance was determined using unpaired two-sided t-test (mean ± SEM). **p-value < 0.01; ns=not significant.

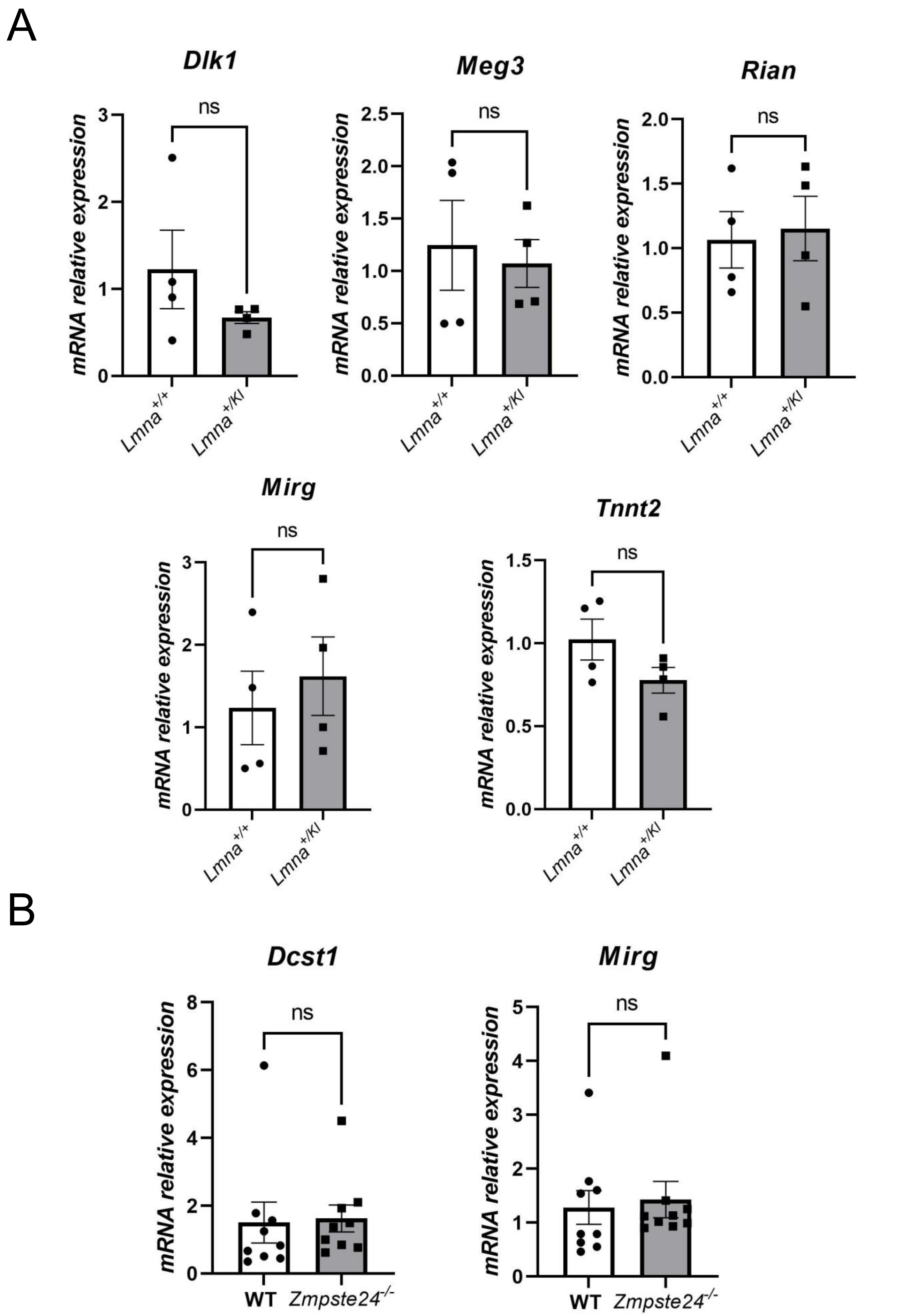

Figure S5. Expression analysis of *Dlk1-Dio3* cluster and related genes in nuclear lamina mutant hearts. **A**, RT-qPCR of selected *Dlk1-Dio3* cluster genes in WT and *Lmna*^{R249W} hearts (refer to Fig. 6A). **B**, RT-qPCR of *Dcst1* and *Mirg* in WT and *Zmpste24*$^{-/-}$ hearts (refer to Fig. 6B). Significance was determined using unpaired two-sided t-test (mean ± SEM). *ns* = not significant.

Supplementary tables:

Table S1. Echocardiographic parameters in WT, *ThPOK*^{KO}, *Chd4*^{KO}, and *dKO* mice. Comprehensive echocardiographic measurements obtained from 5- and 10-week-old mice of each genotype. The table includes all individual values for males and females, with mouse identification codes provided; male animals are indicated in bold, and females in regular font.

Table S2. Differential gene expression from bulk RNA-seq analysis. Differentially expressed genes (DEGs) identified in *ThPOK*^{KO}, *Chd4*^{KO} and *dKO* mouse hearts compared to WT littermates. Additional comparisons between *Chd4*^{KO} vs *ThPOK*^{KO} and *Chd4*^{KO} vs *dKO* are included. Differential expression was determined using the Wilcoxon test with Bonferroni-adjusted p-values. Average Log2 fold changes for each DEG are reported.
